# Supplementary material for: Synthesis of Substituted 1,2-Dihydroisoquinolines by Palladium-Catalyzed Cascade Cyclization–Coupling of Trisubstituted Allenamides with Arylboronic Acids
Source: Molecules. 2024 Jun 19;29(12):2917. doi: 10.3390/molecules29122917 (PMC11206658; doi:10.3390/molecules29122917)

Supporting Information for

Synthesis of Substituted 1,2-dihydroisoquinolines by Palladium-Catalyzed Cascade Cyclization–Coupling of Trisubstituted Allenamides with Arylboronic Acids

Masahiro Yoshida*, Ryunosuke Imaji and Shinya Shiomi

Faculty of Pharmaceutical Sciences, Tokushima Bunri University, 180 Nishihamabouji, Yamashiro-cho, Tokushima, 770-8514, Japan.

E-mail: yoshida@ph.bunri-u.ac.jp

List of Contents

^{1}H-NMR and ^{13}C-NMR Spectra SI-2–SI-22

Compound **1a**, ^{1}H-NMR (500 MHz, $CDCl_3$)

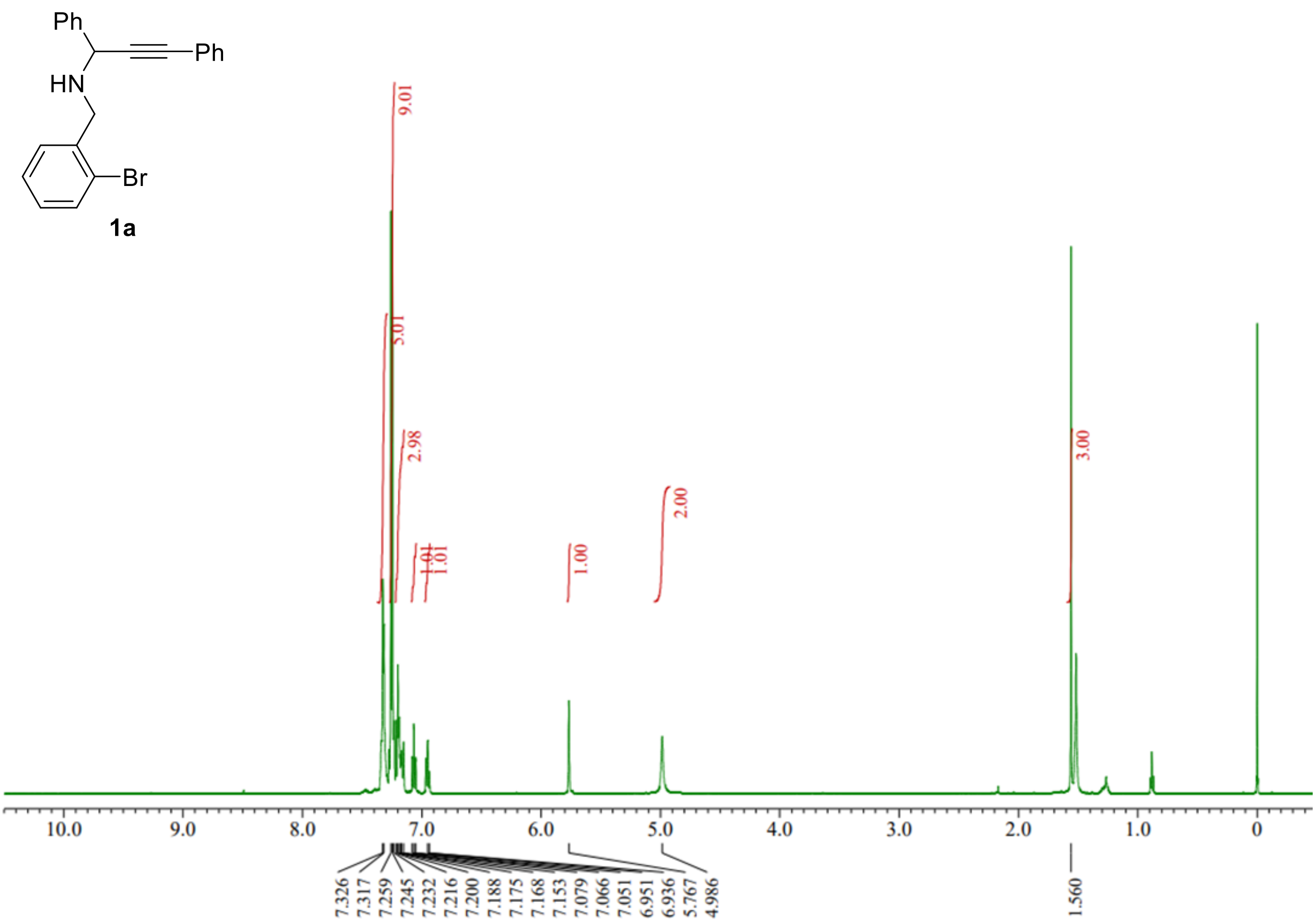

^{13}C-NMR (125 MHz, $CDCl_3$)

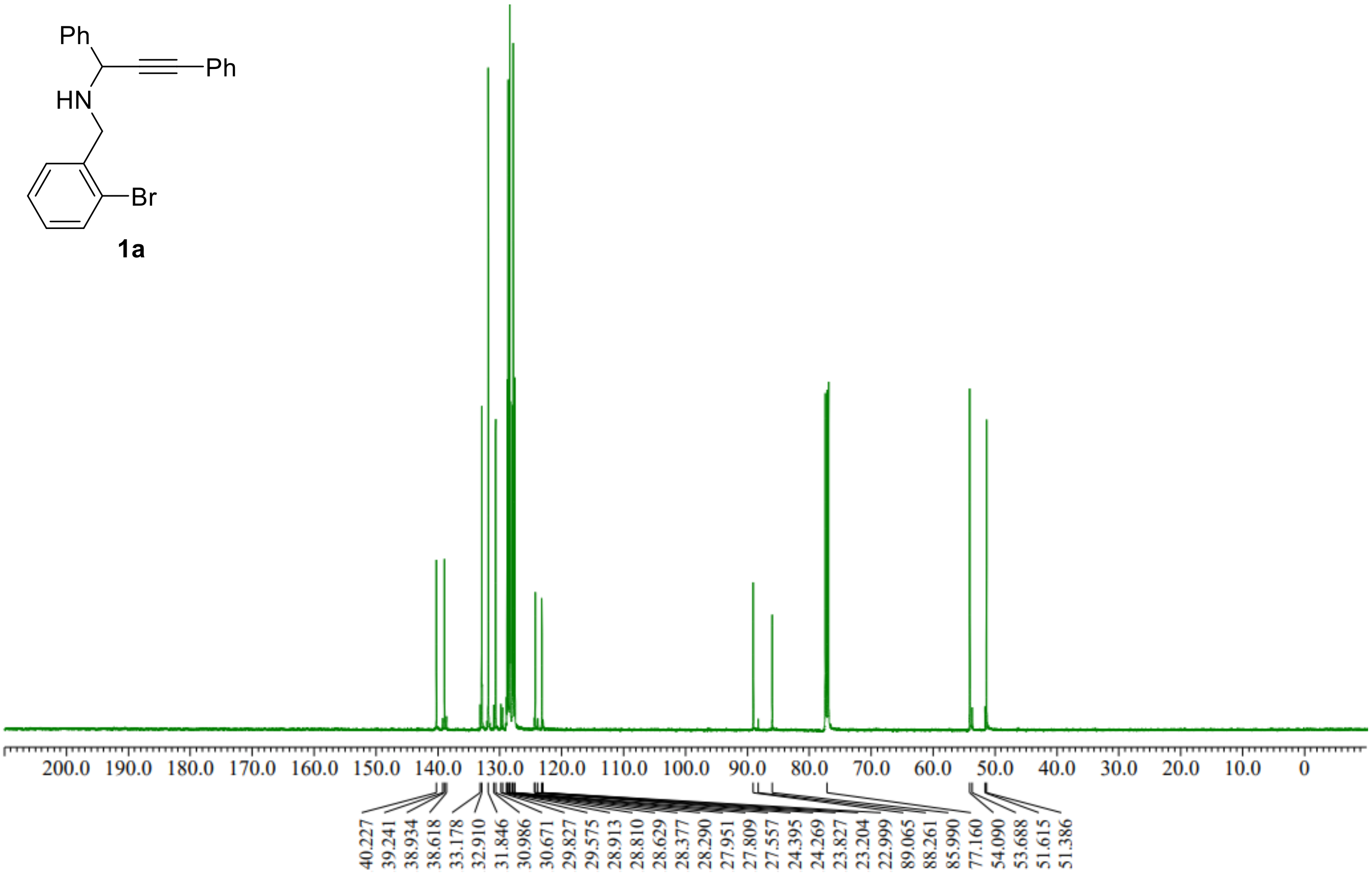

Compound **1b**, ^{1}H-NMR (500 MHz, $CDCl_3$)

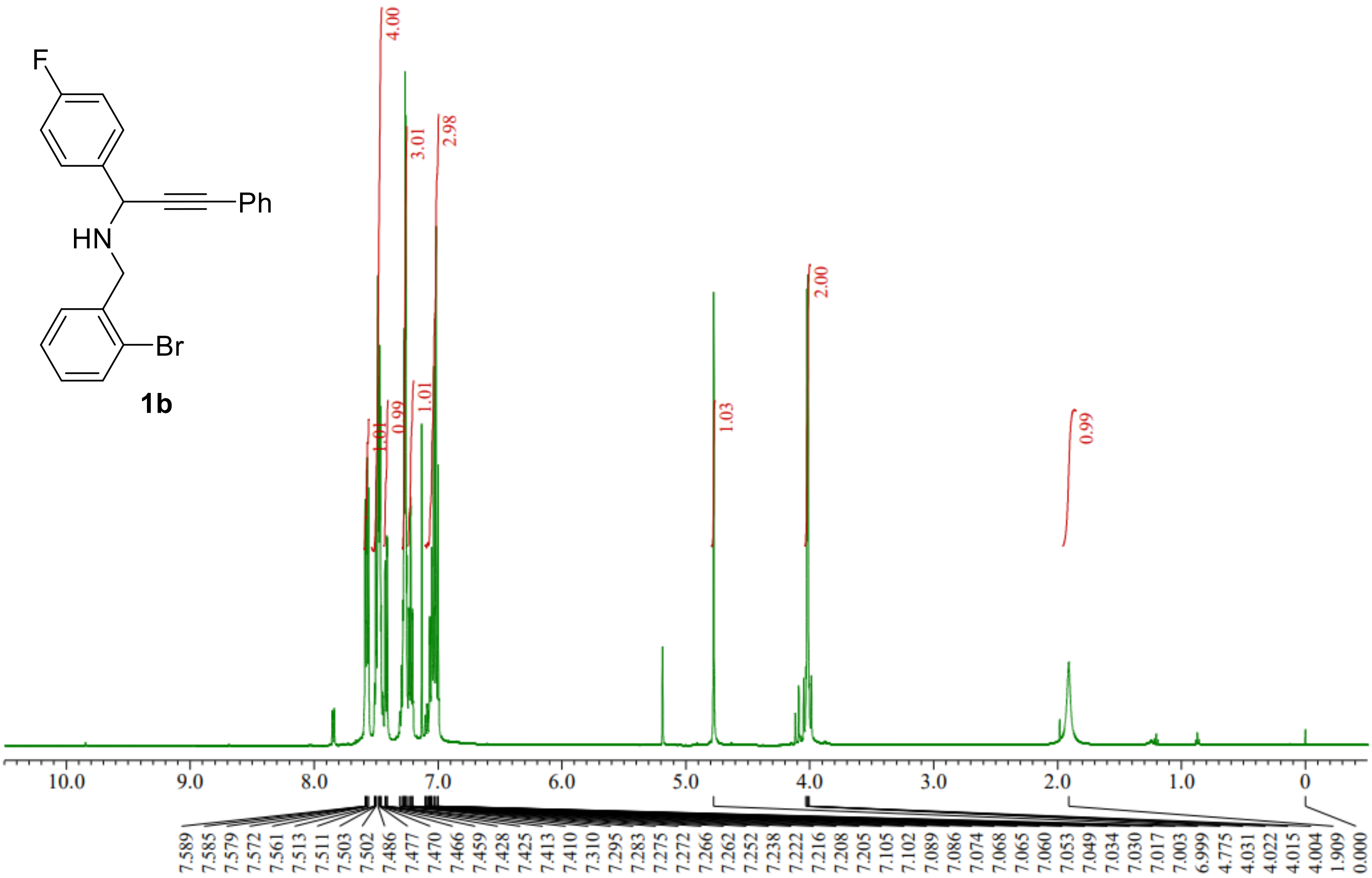

^{13}C-NMR (125 MHz, $CDCl_3$)

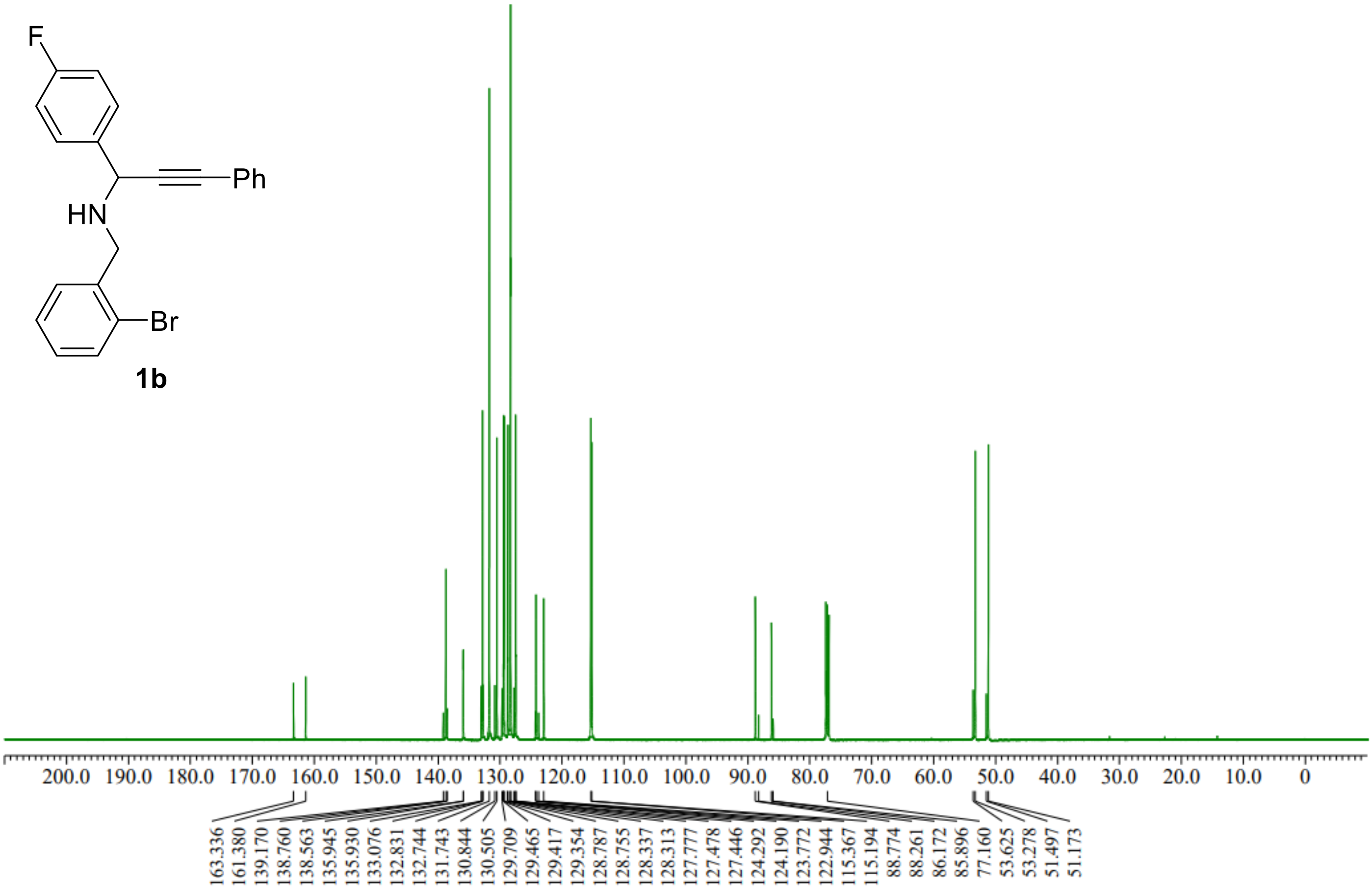

Compound **1c**, ^{1}H-NMR (500 MHz, $CDCl_3$)

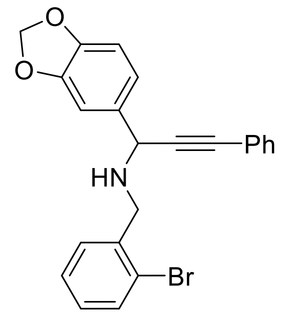

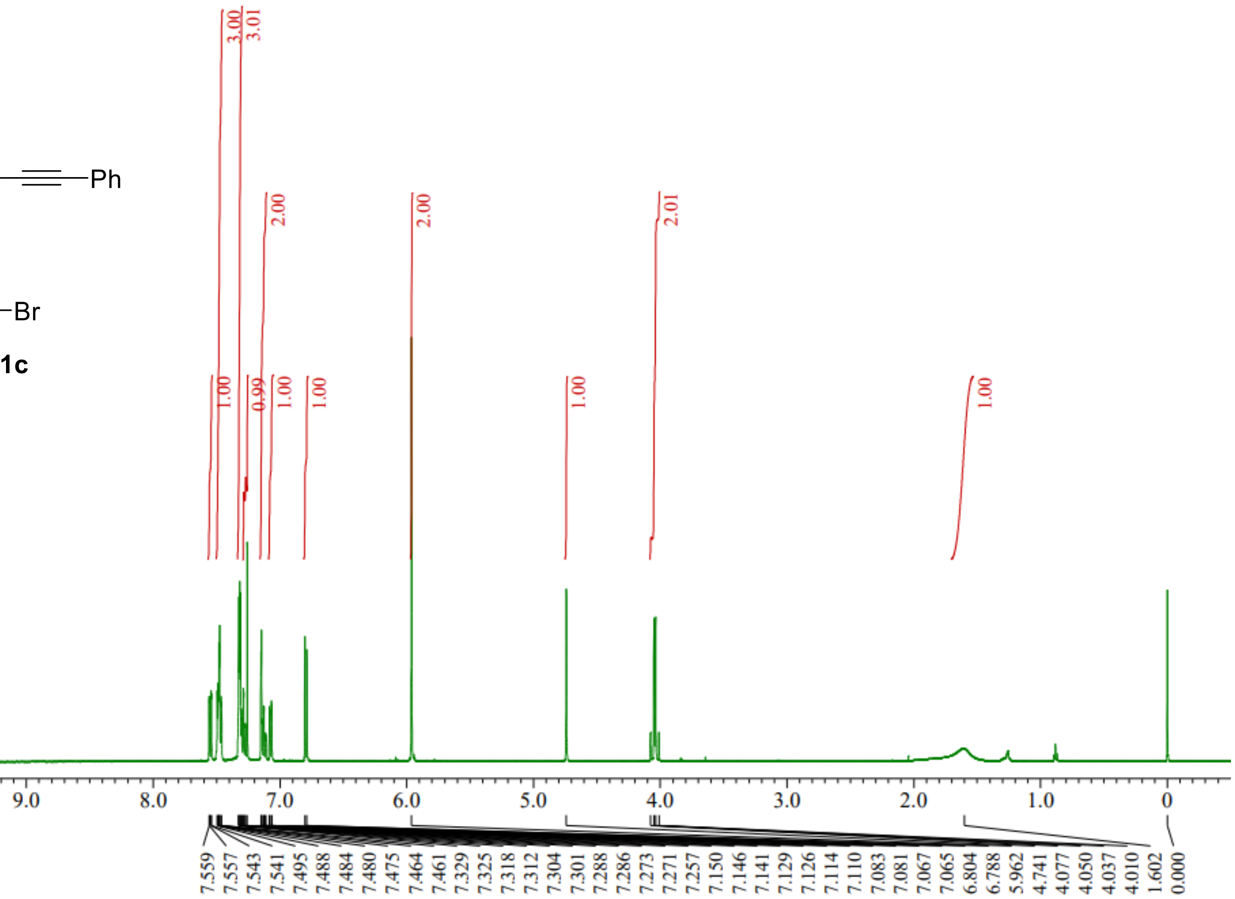

^{13}C-NMR (125 MHz, $CDCl_3$)

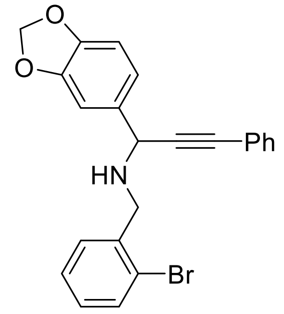

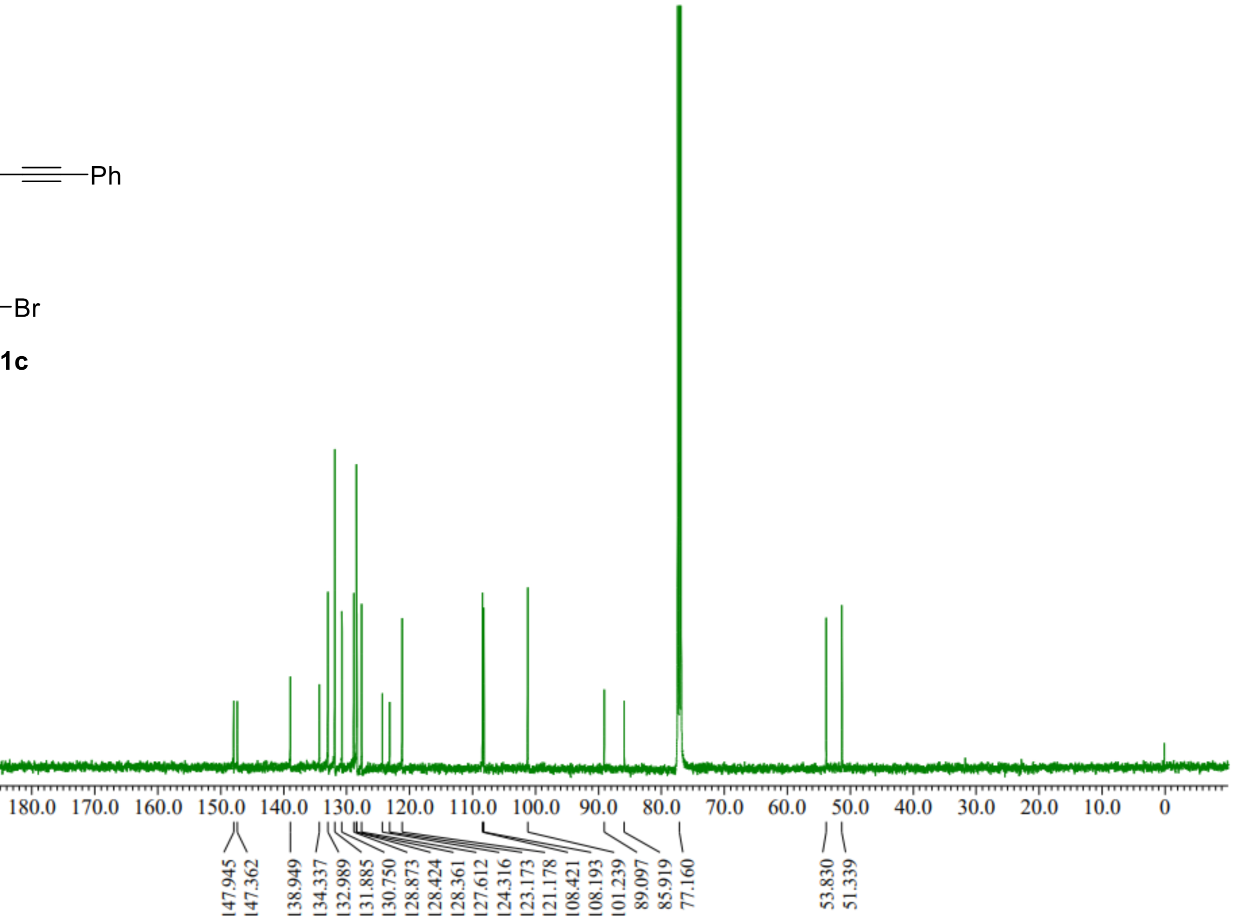

Compound **1d**, ^{1}H-NMR (500 MHz, $CDCl_3$)

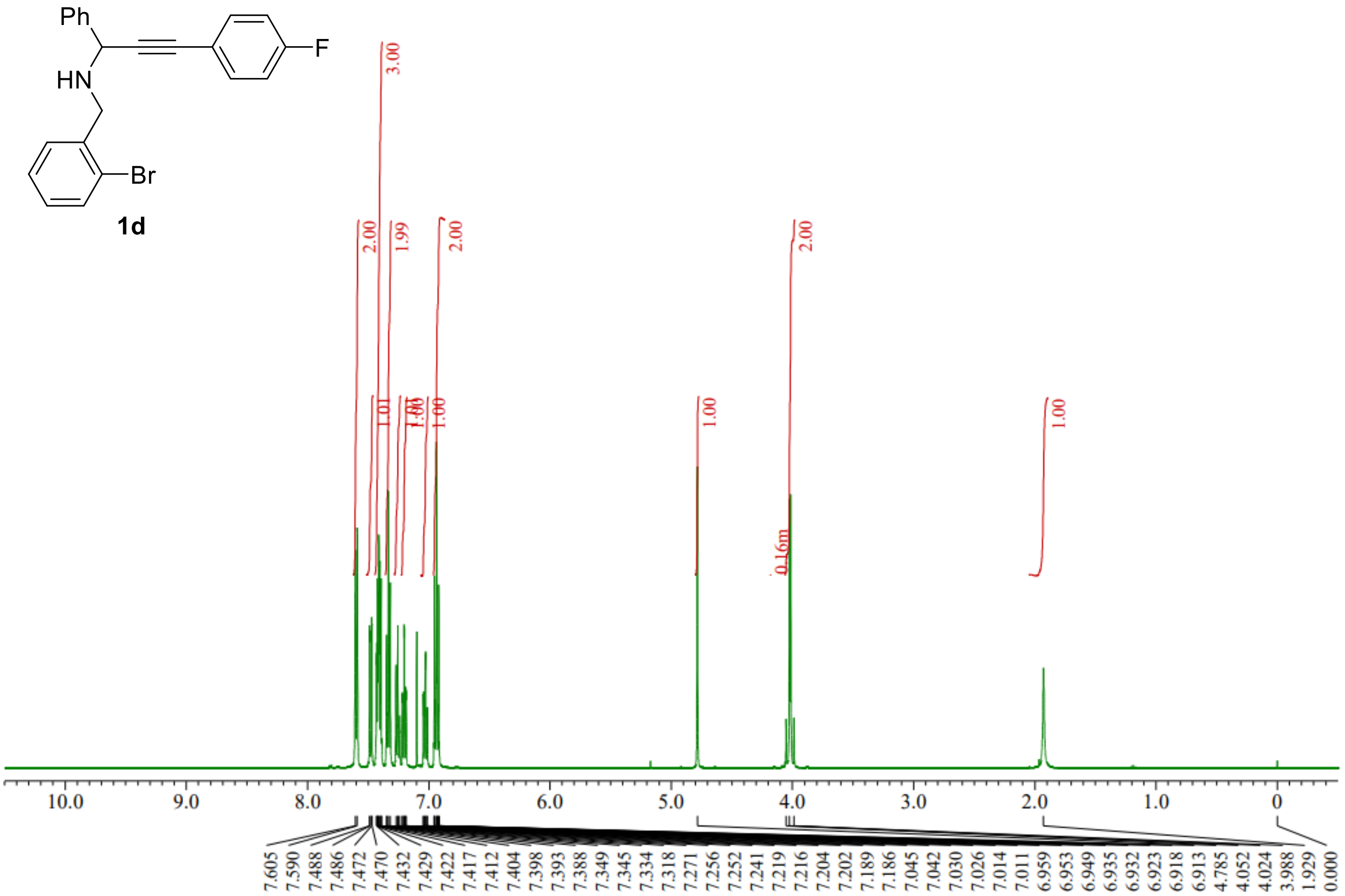

^{13}C-NMR (125 MHz, $CDCl_3$)

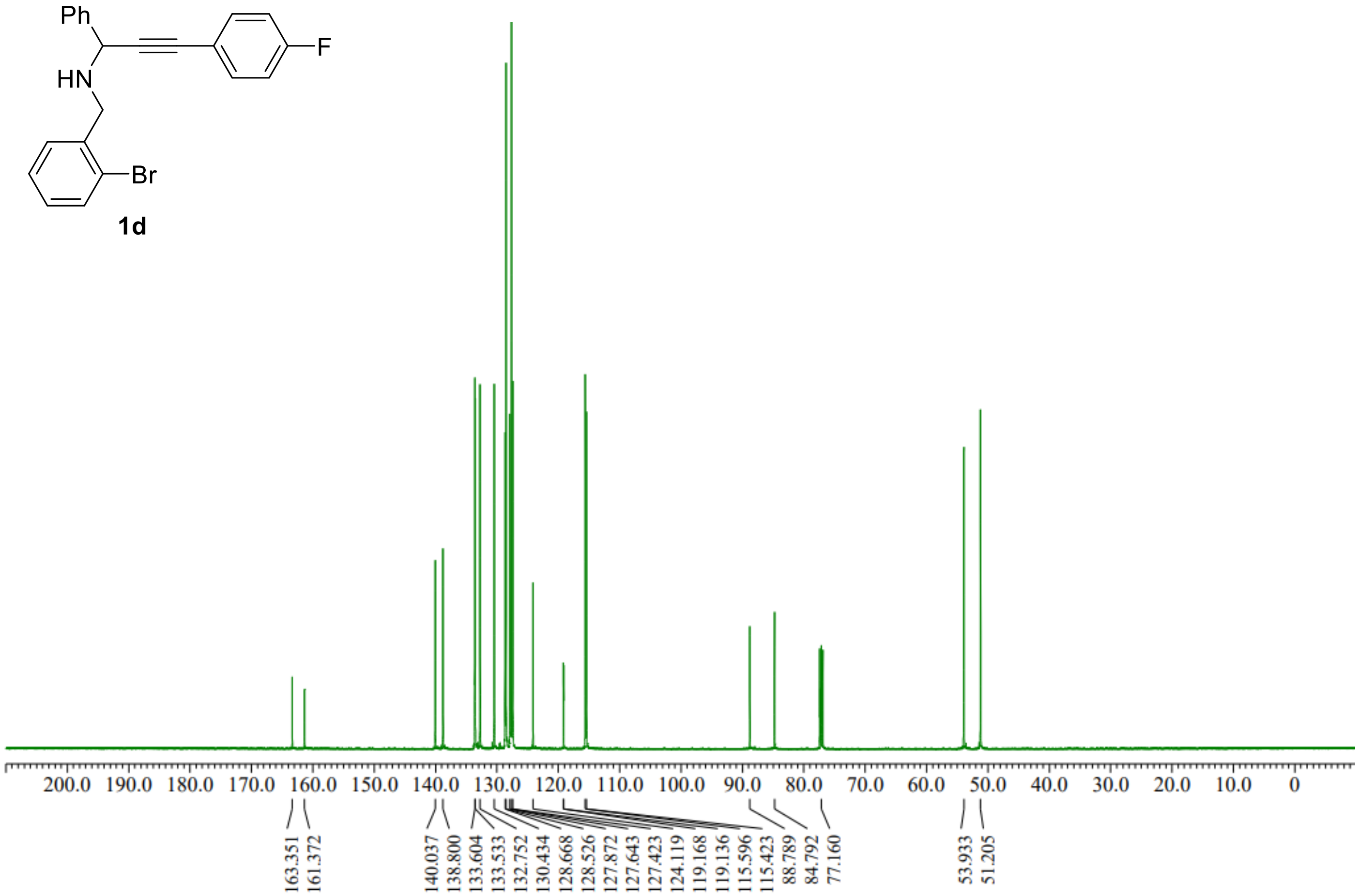

Compound **1e**, ^{1}H-NMR (500 MHz, $CDCl_3$)

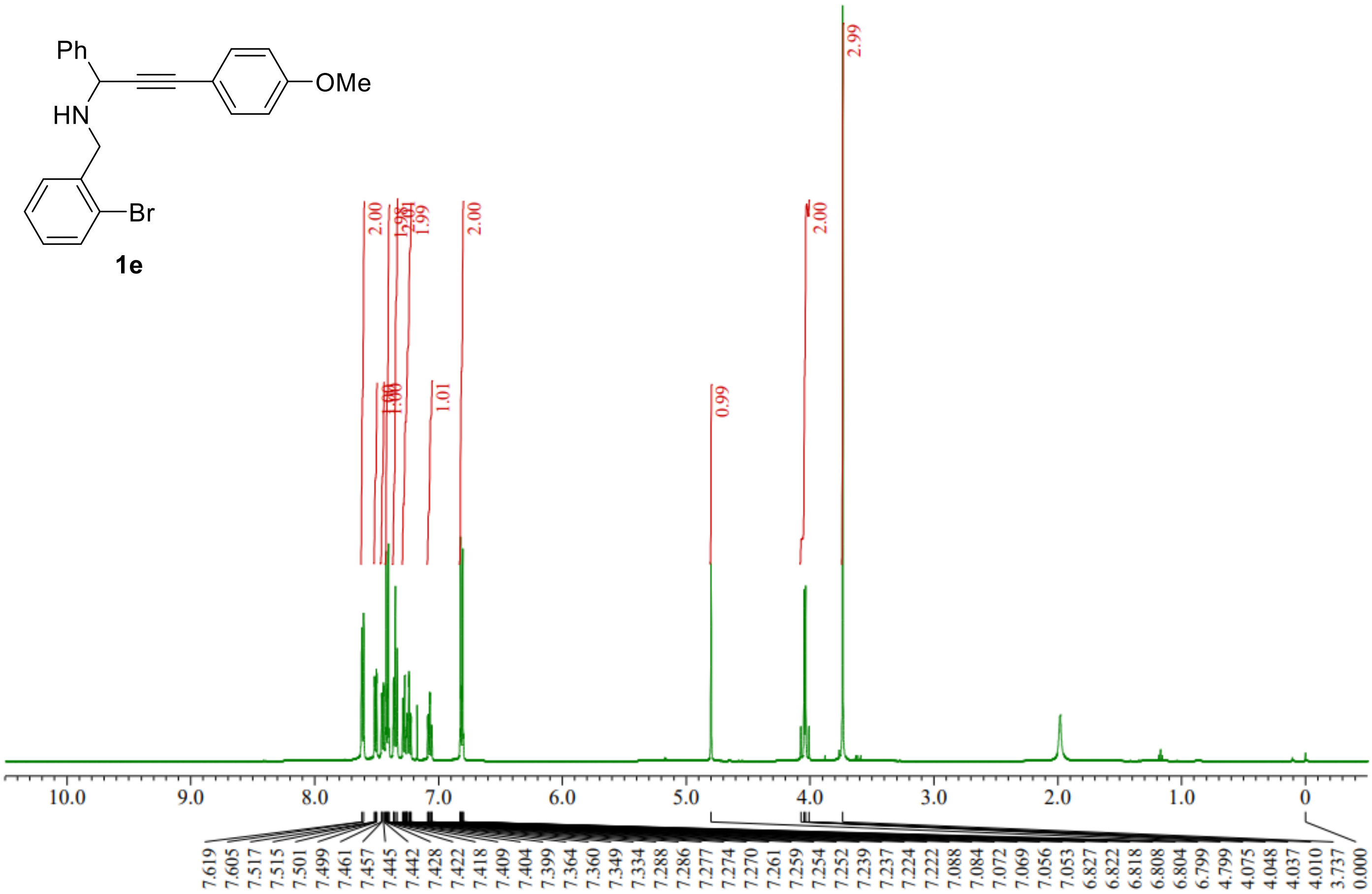

^{13}C-NMR (125 MHz, $CDCl_3$)

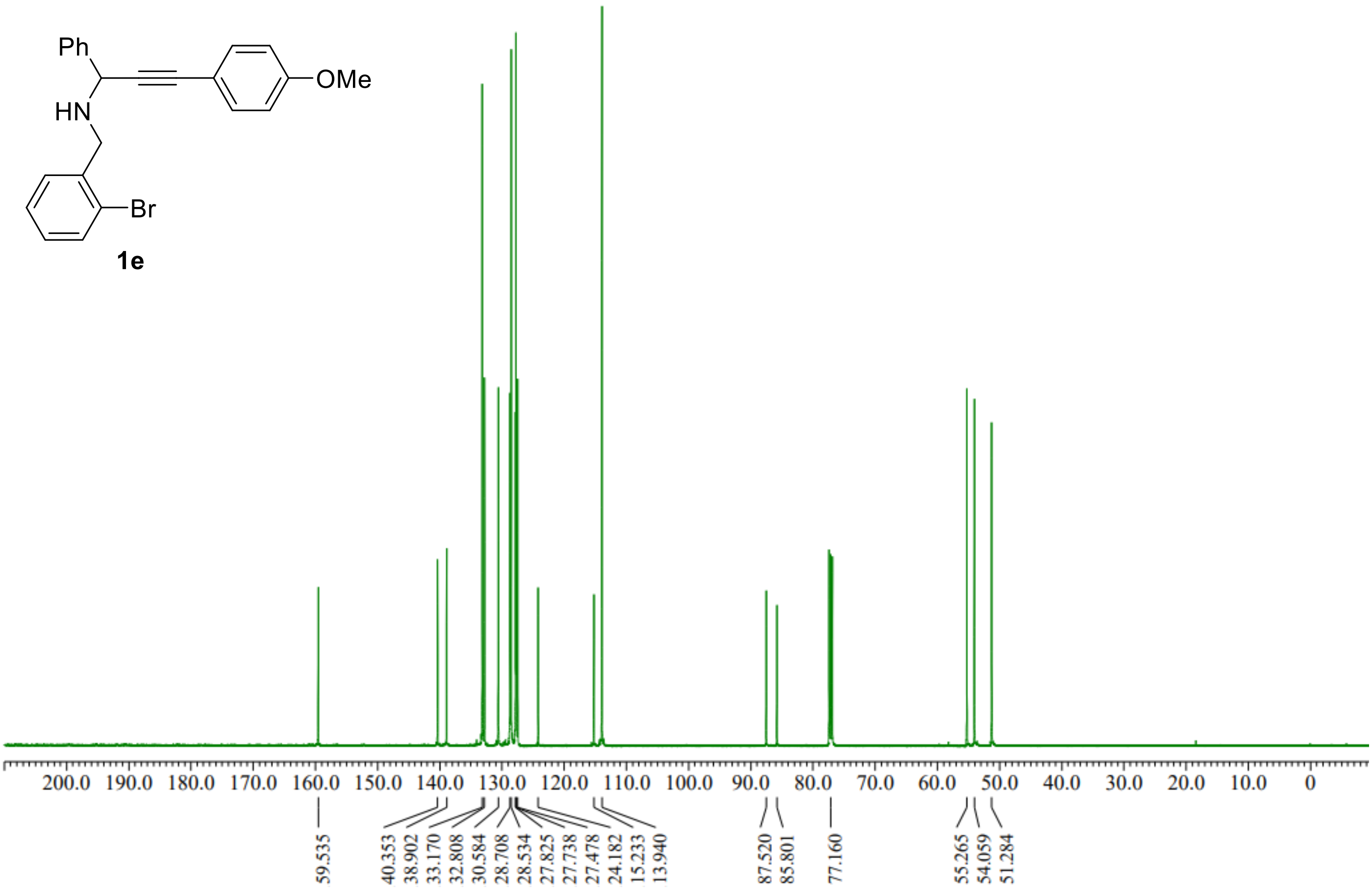

Compound **2a**, ^{1}H-NMR (500 MHz, $CDCl_3$)

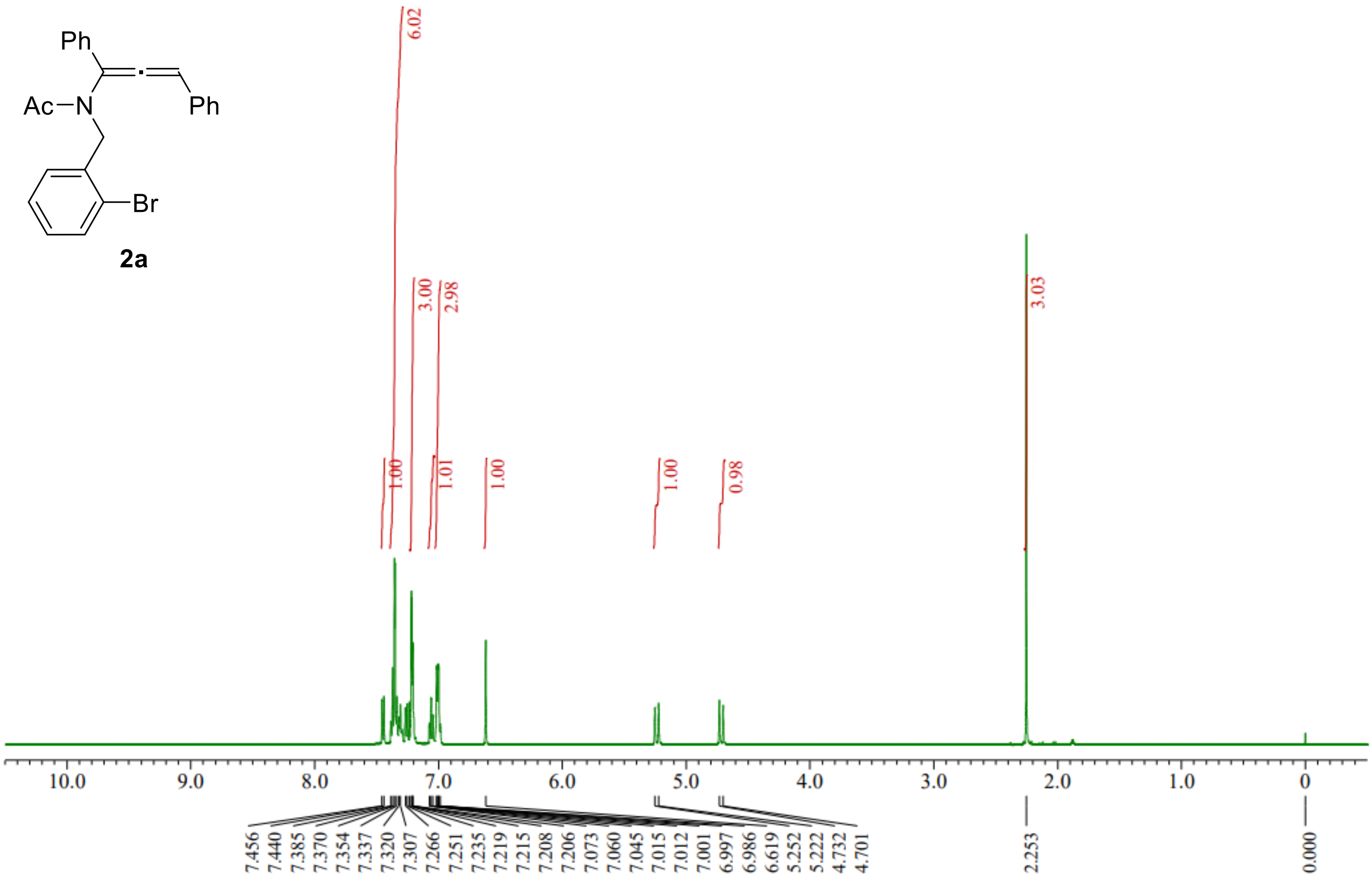

^{13}C-NMR (125 MHz, $CDCl_3$)

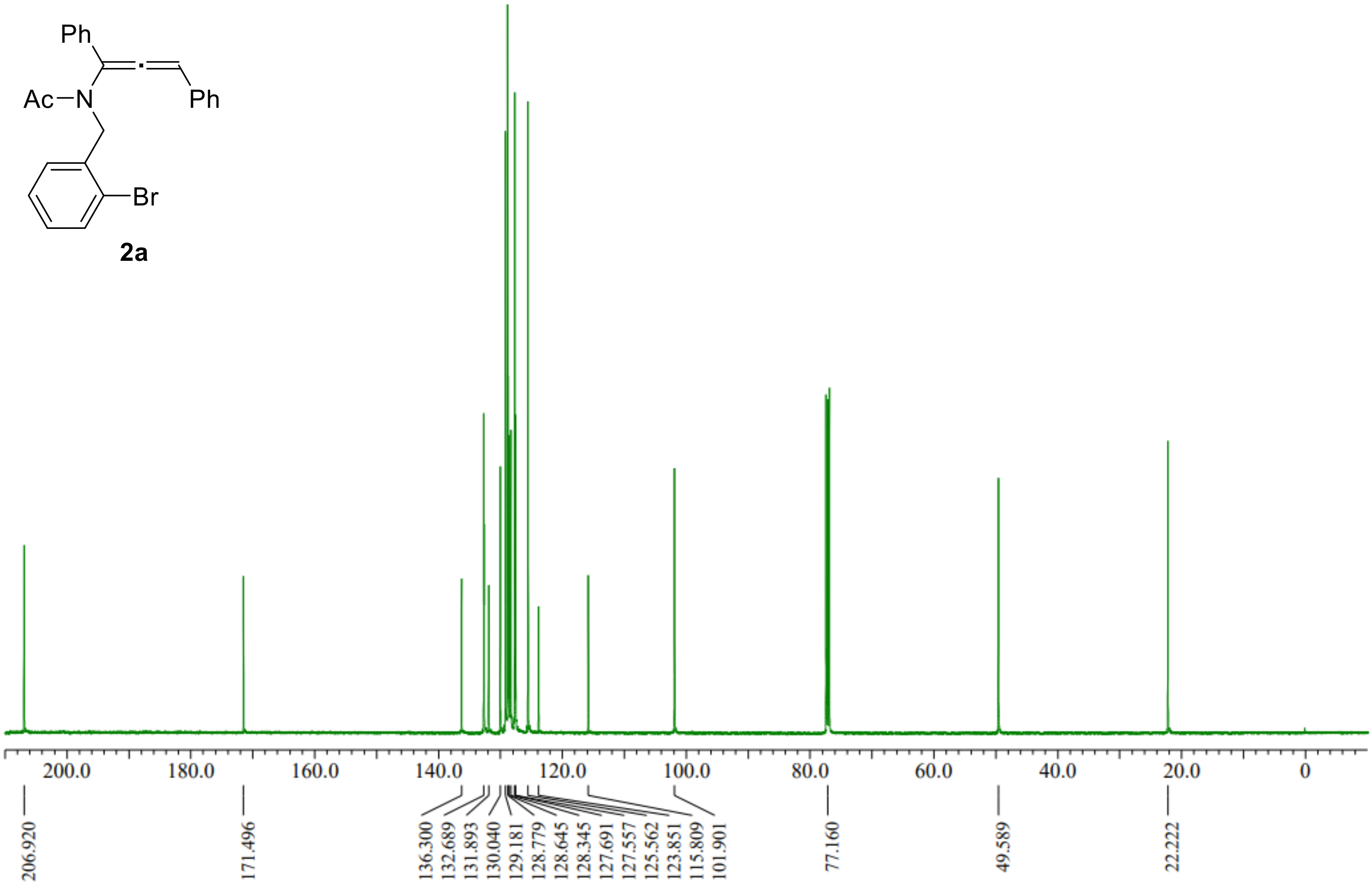

Compound **2b**, ^{1}H-NMR (500 MHz, $CDCl_3$)

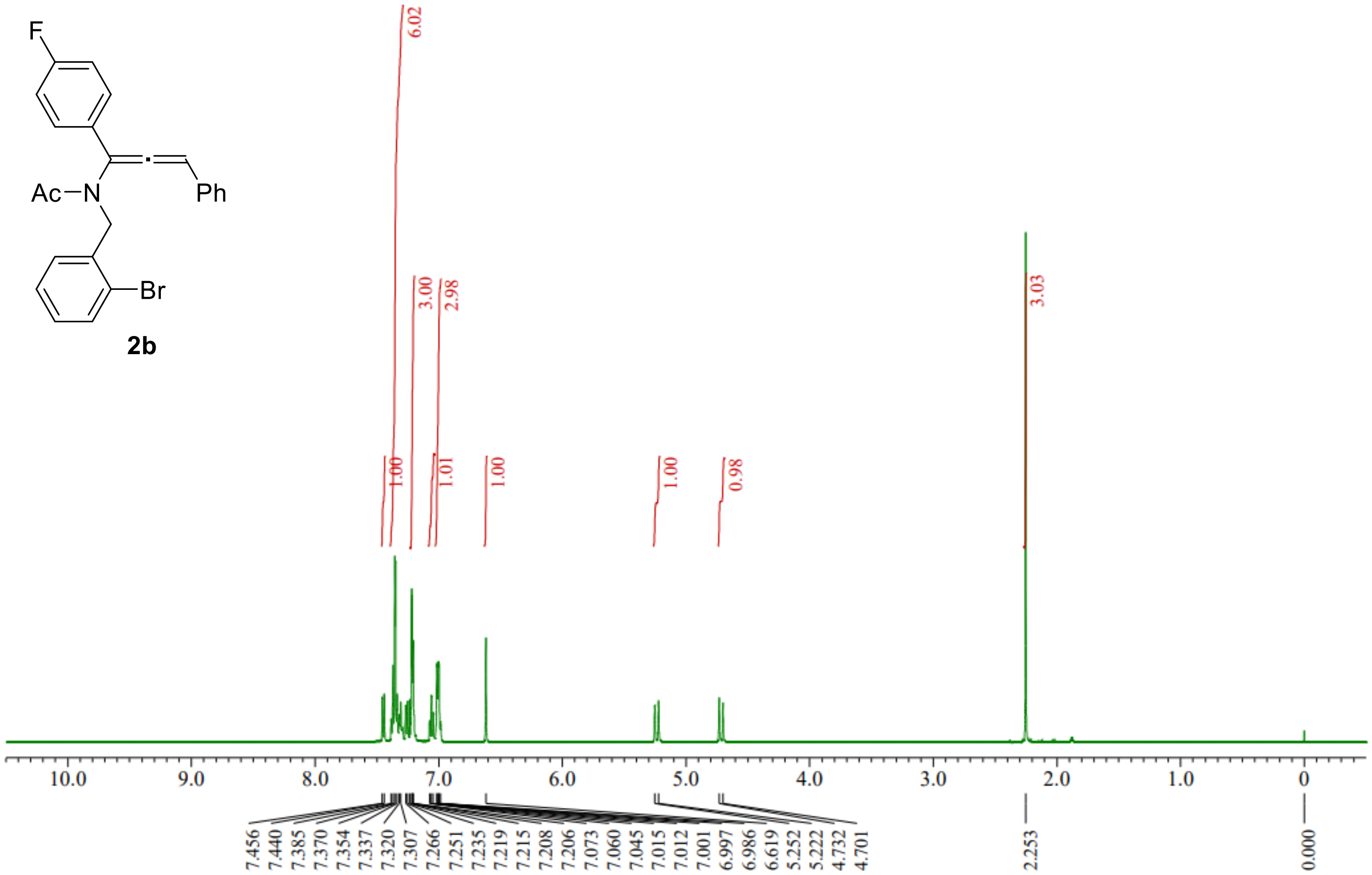

^{13}C-NMR (125 MHz, $CDCl_3$)

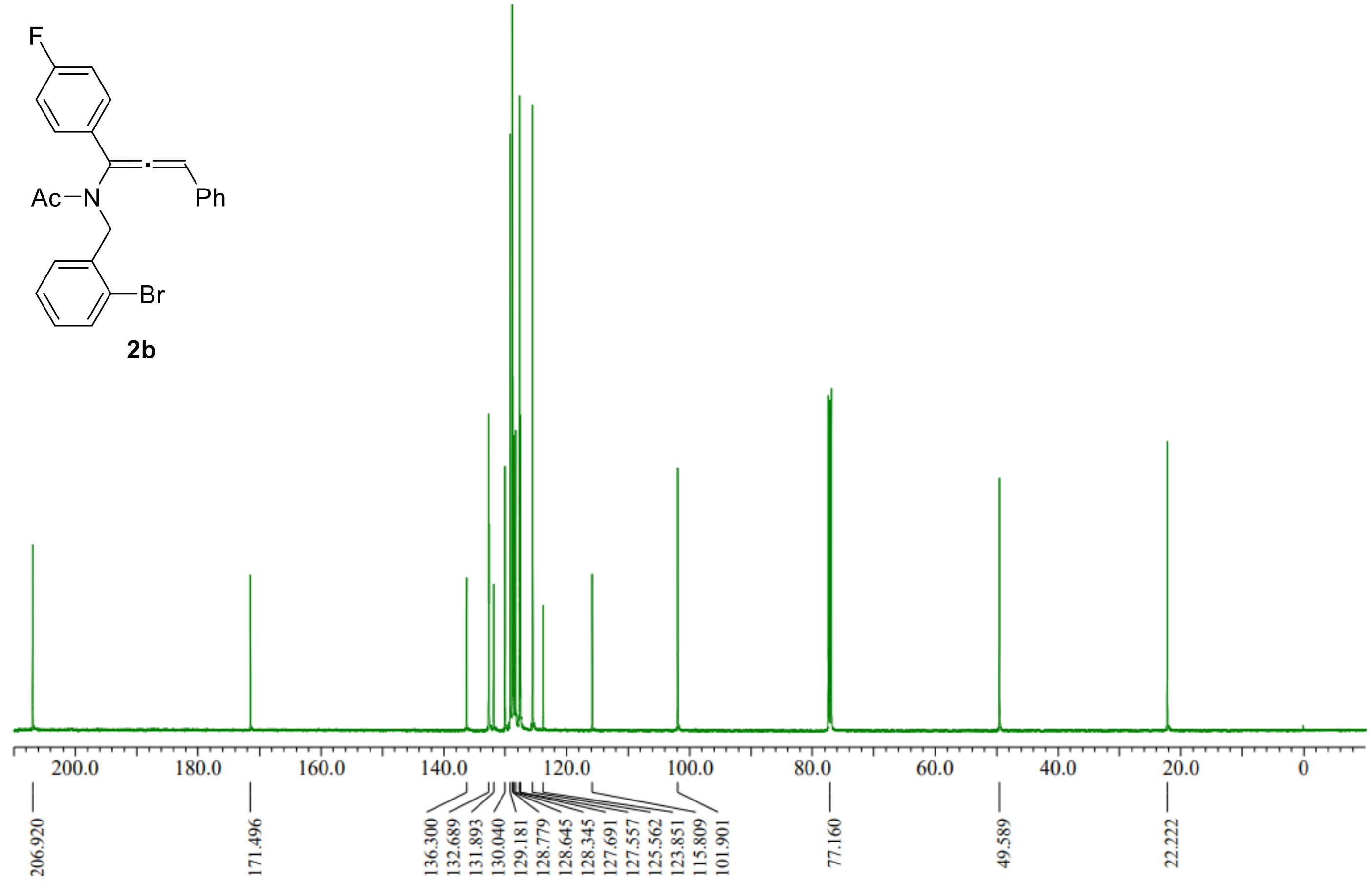

Compound **2c**, ^{1}H-NMR (500 MHz, $CDCl_3$)

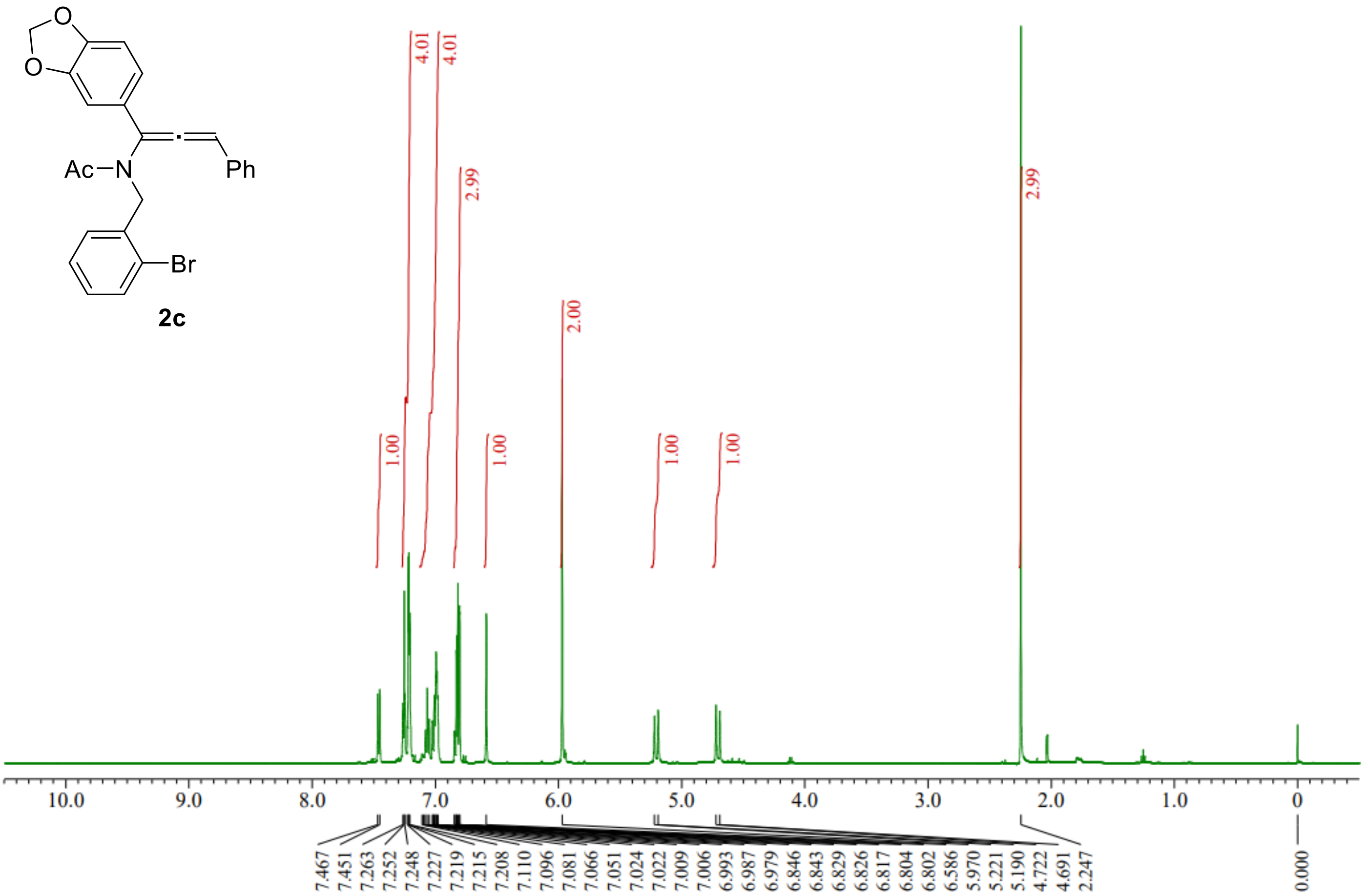

^{13}C-NMR (125 MHz, $CDCl_3$)

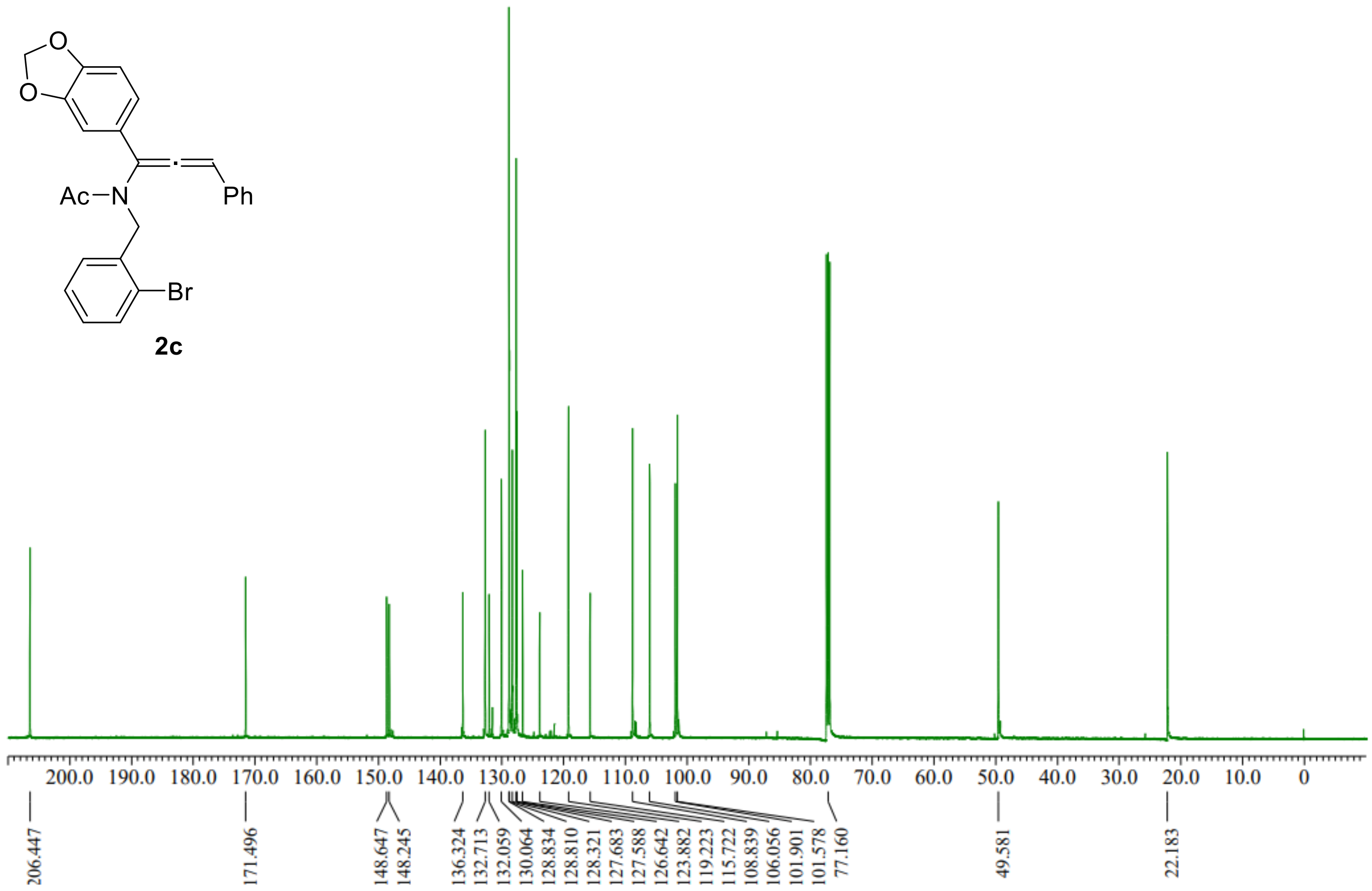

Compound **2d**, ^{1}H-NMR (500 MHz, $CDCl_3$)

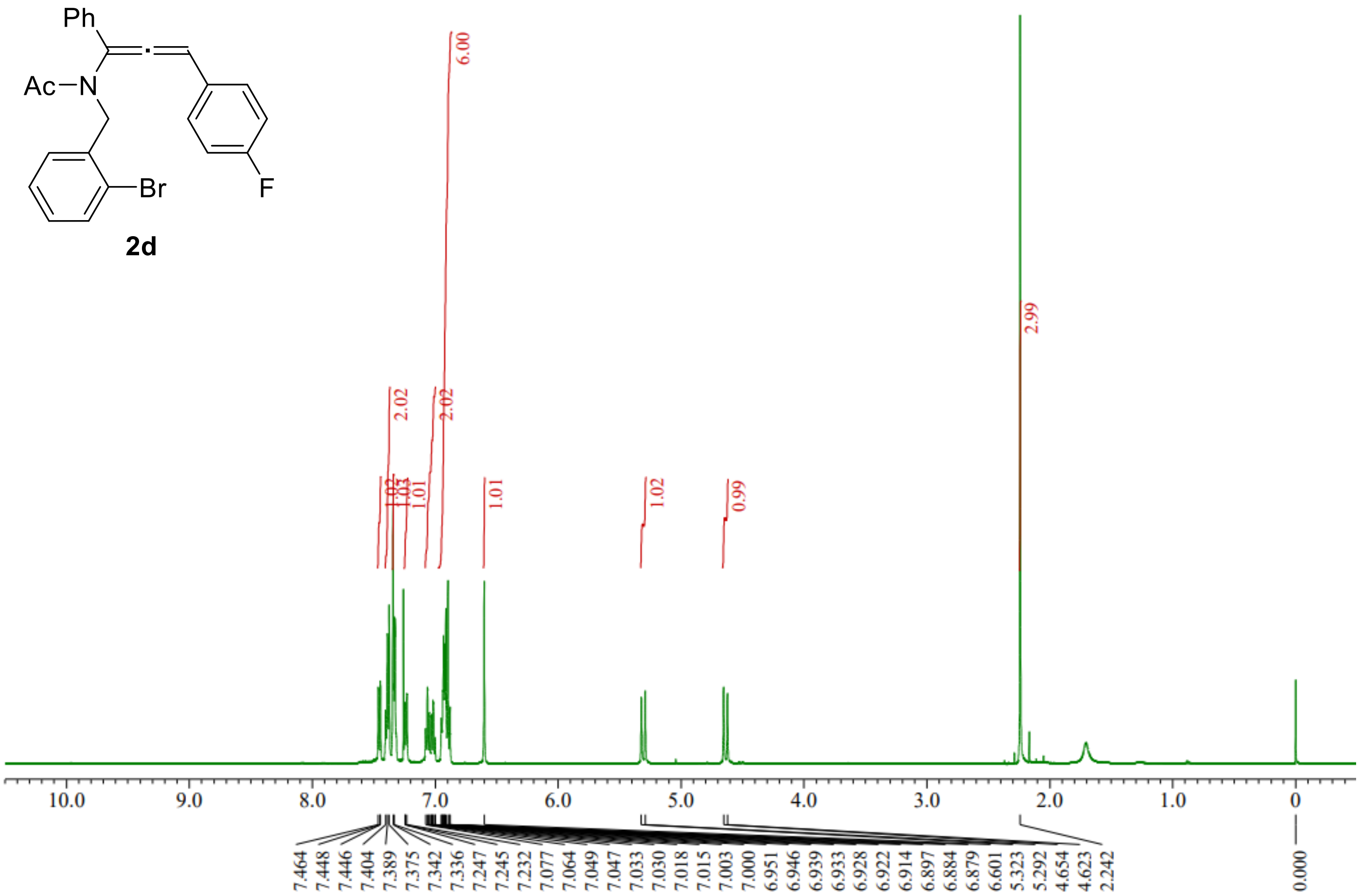

^{13}C-NMR (125 MHz, $CDCl_3$)

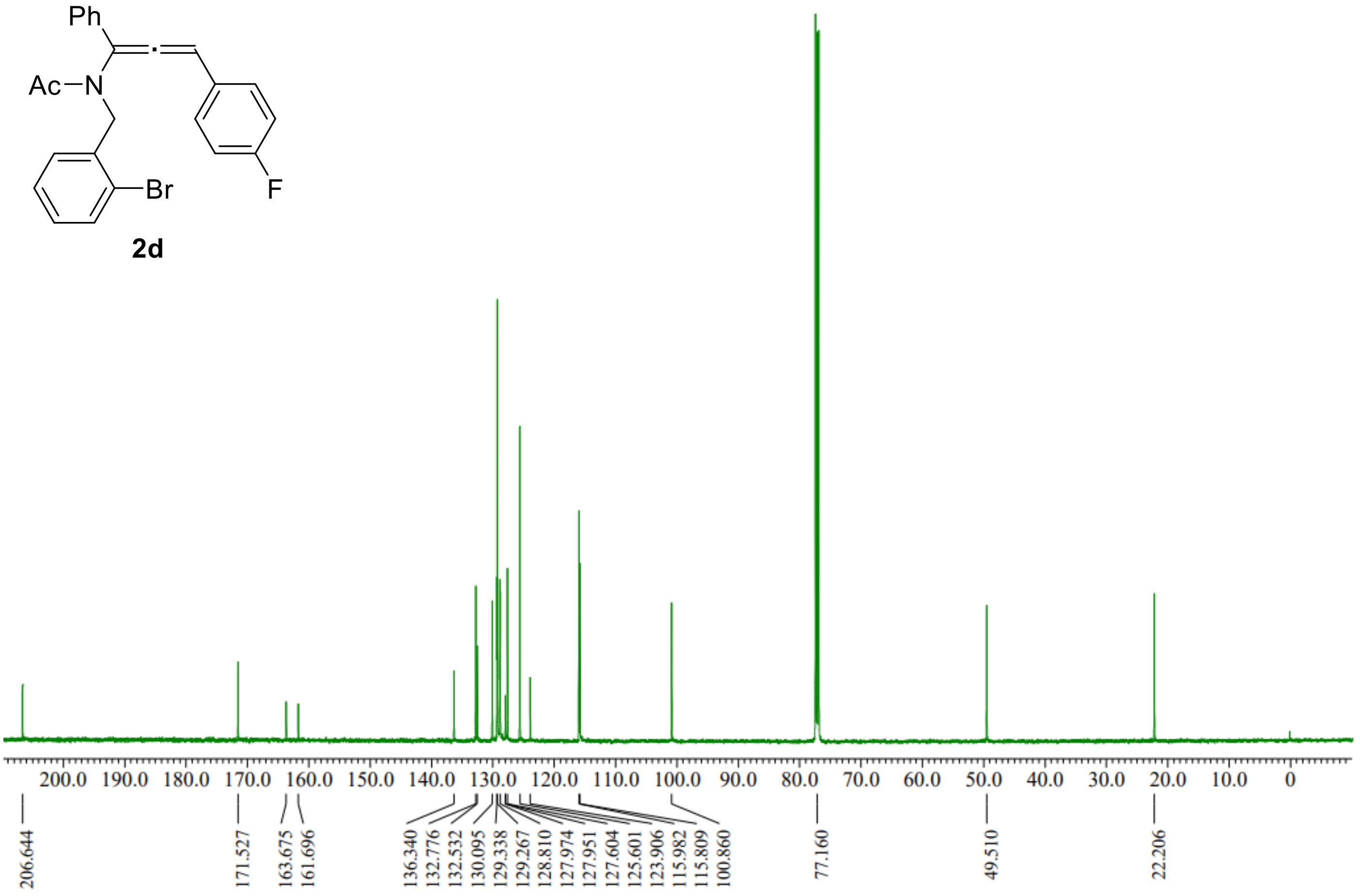

Compound **2e**, ^{1}H-NMR (500 MHz, $CDCl_3$)

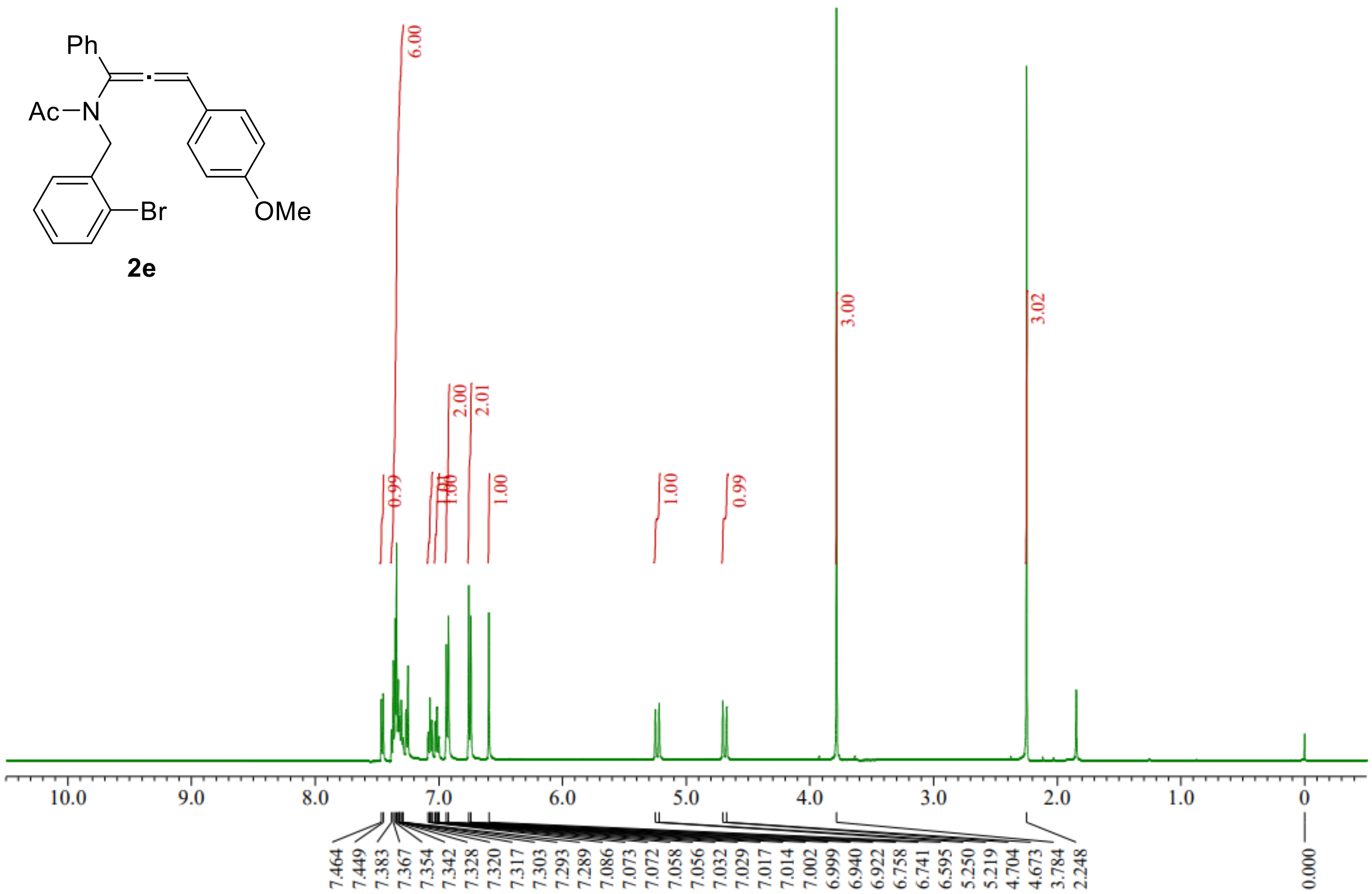

^{13}C-NMR (125 MHz, $CDCl_3$)

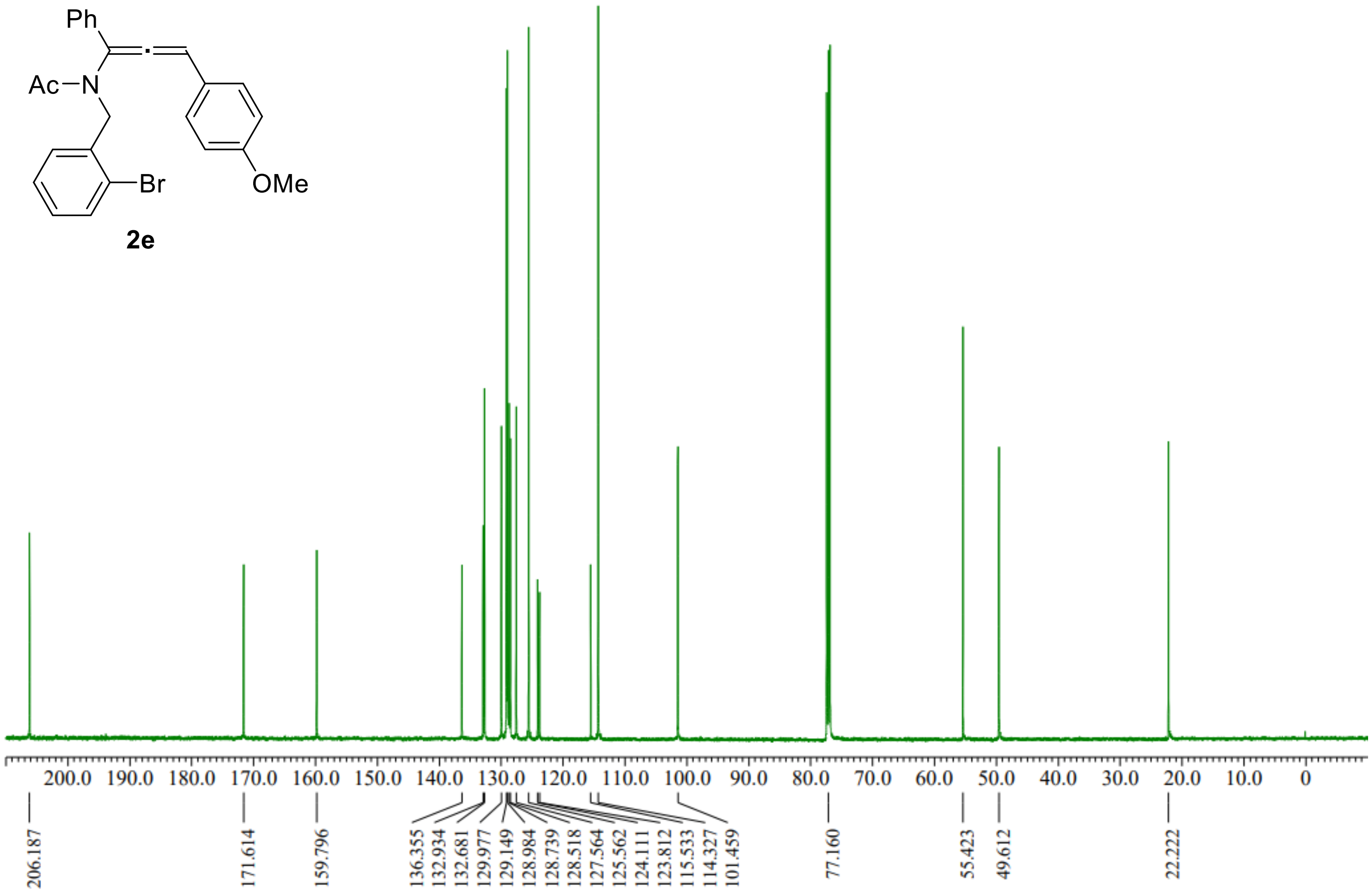

Compound **4aa**, ^{1}H-NMR (500 MHz, $CDCl_3$)

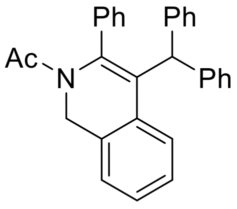

4aa

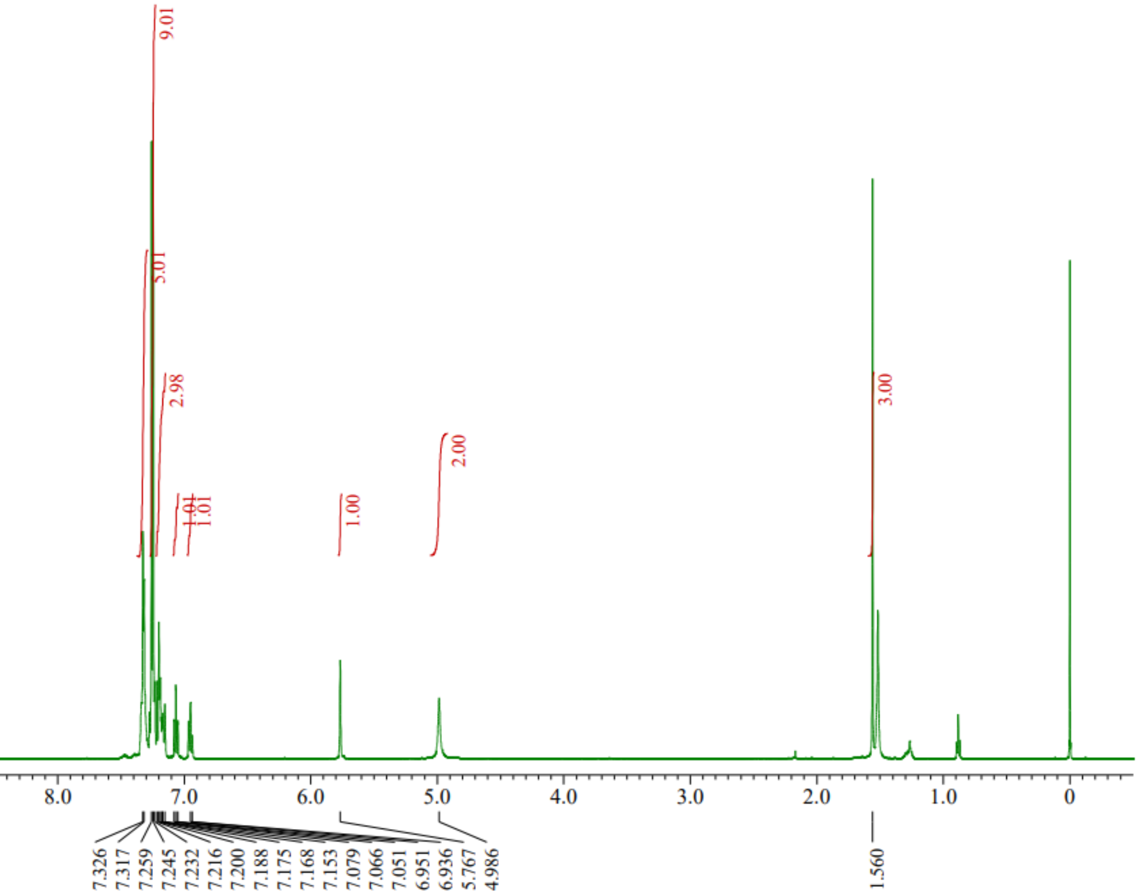

^{13}C-NMR (125 MHz, $CDCl_3$)

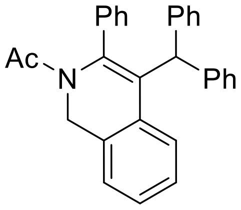

4aa

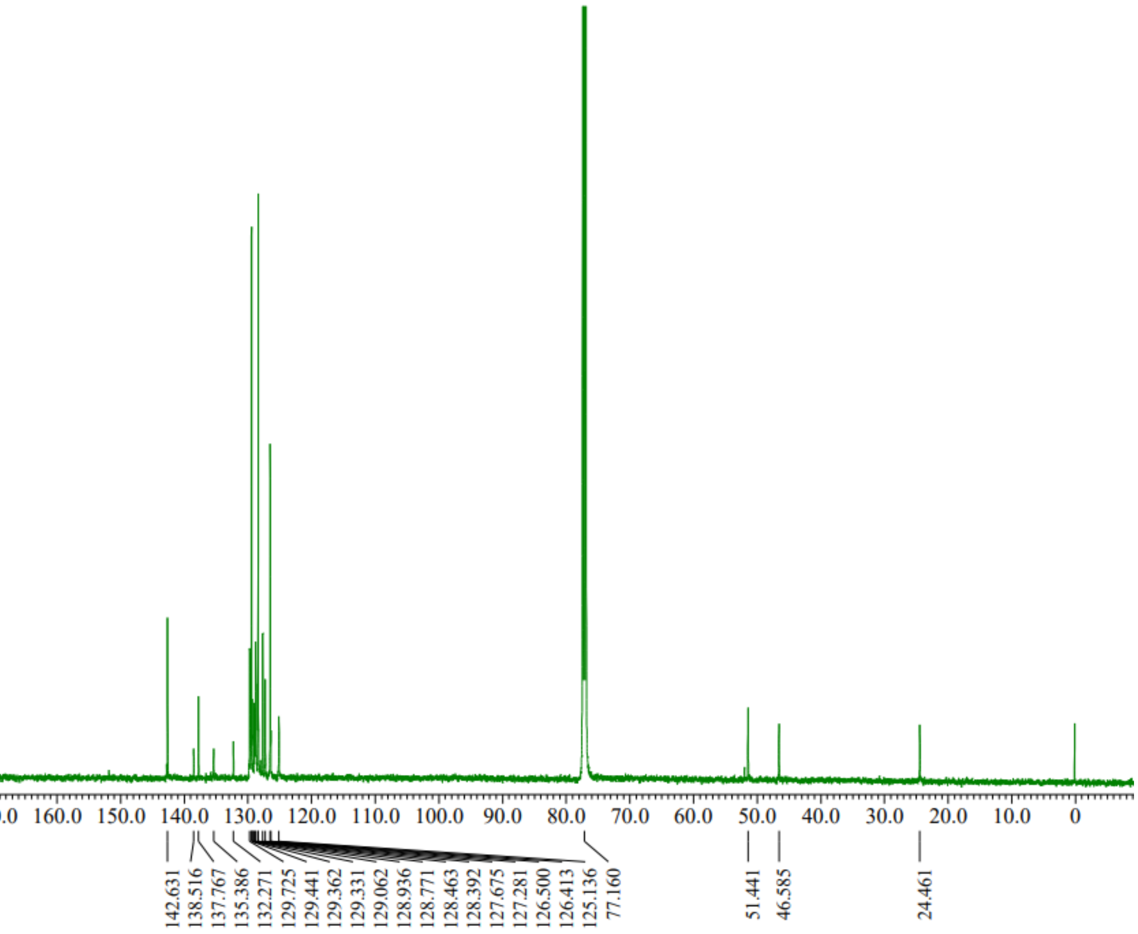

Compound **4ab(4ea)**, ^{1}H-NMR (500 MHz, $CDCl_3$)

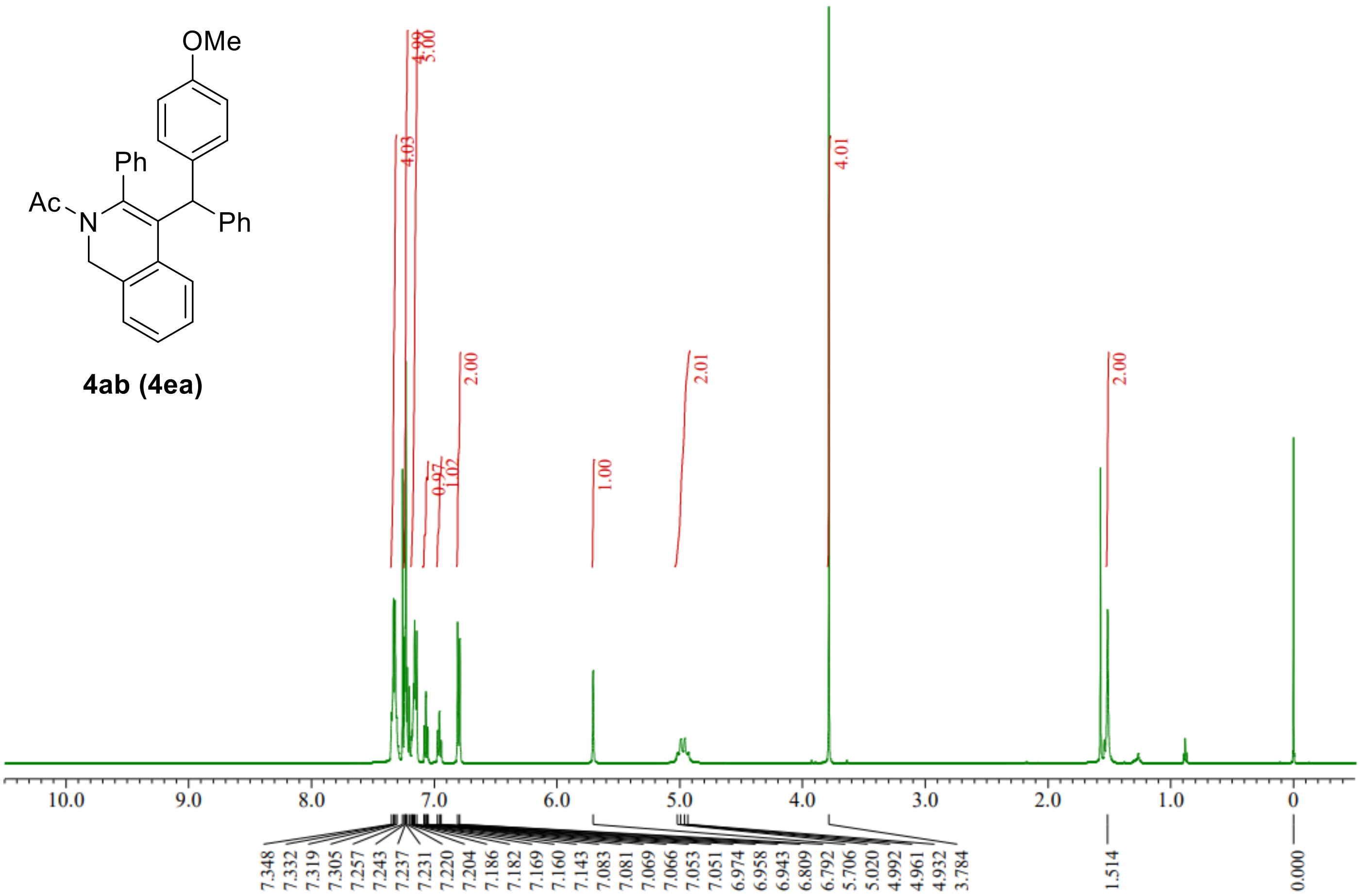

^{13}C-NMR (125 MHz, $CDCl_3$)

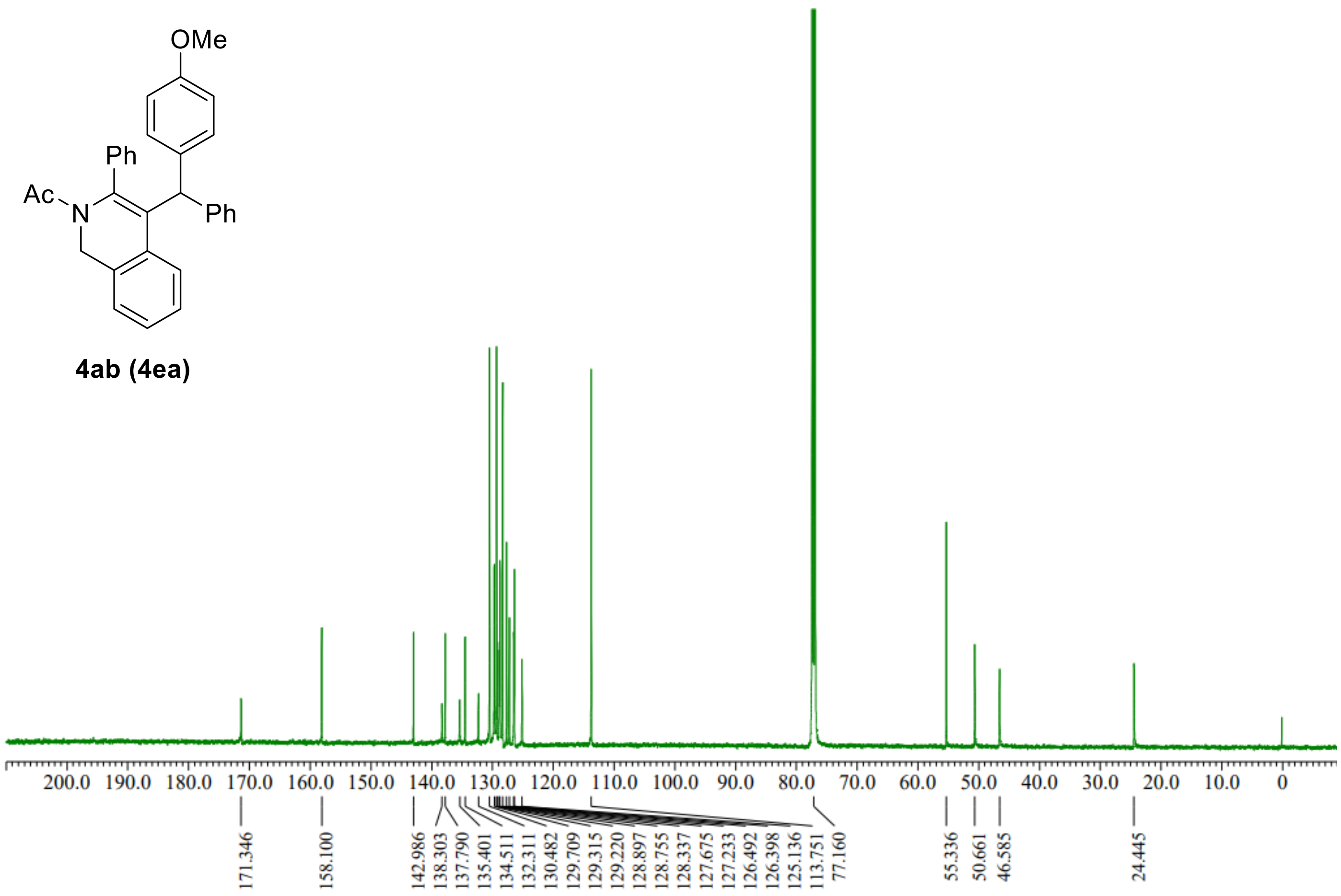

Compound **4ac**, ^{1}H-NMR (500 MHz, $CDCl_3$)

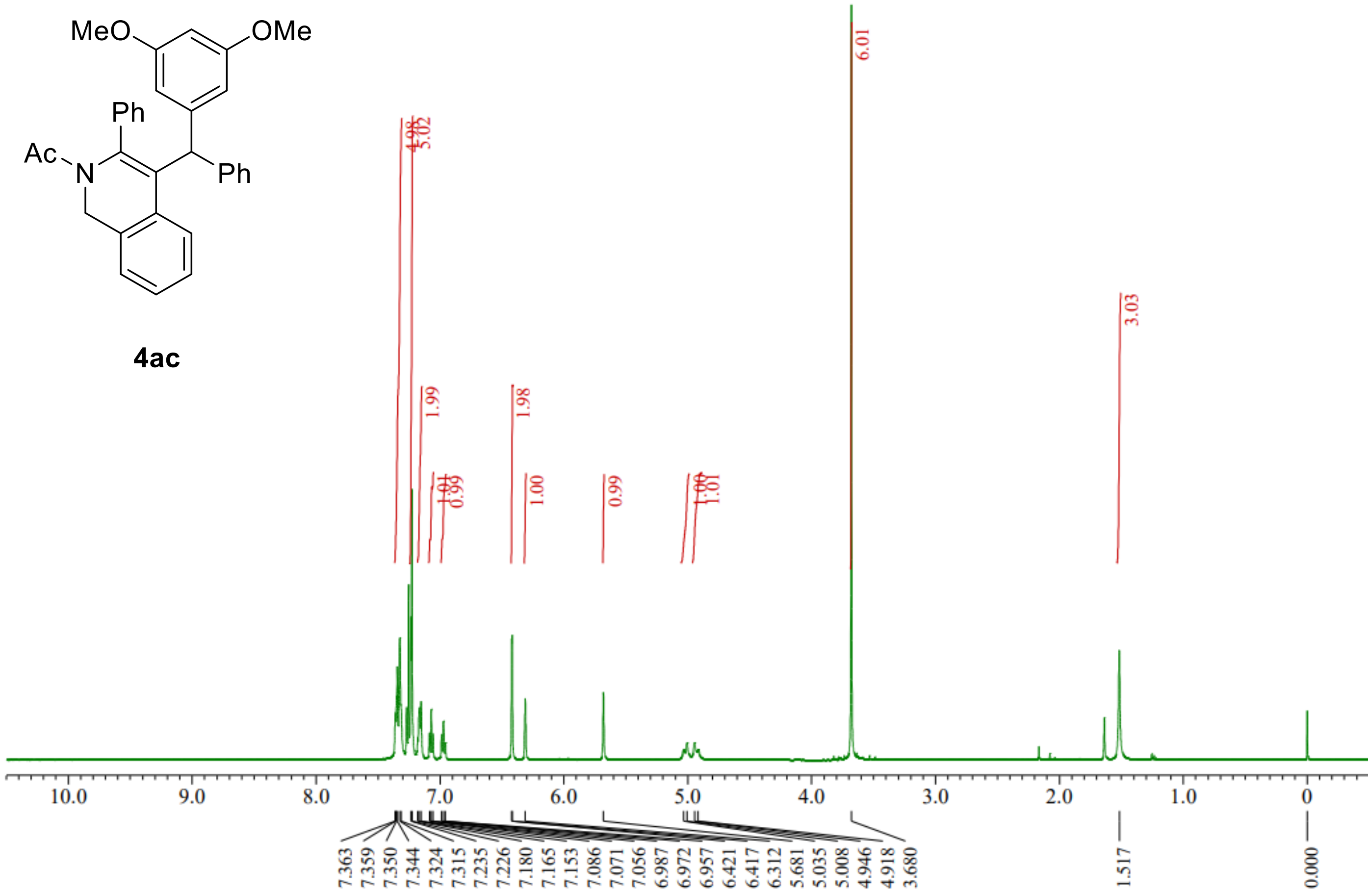

^{13}C-NMR (125 MHz, $CDCl_3$)

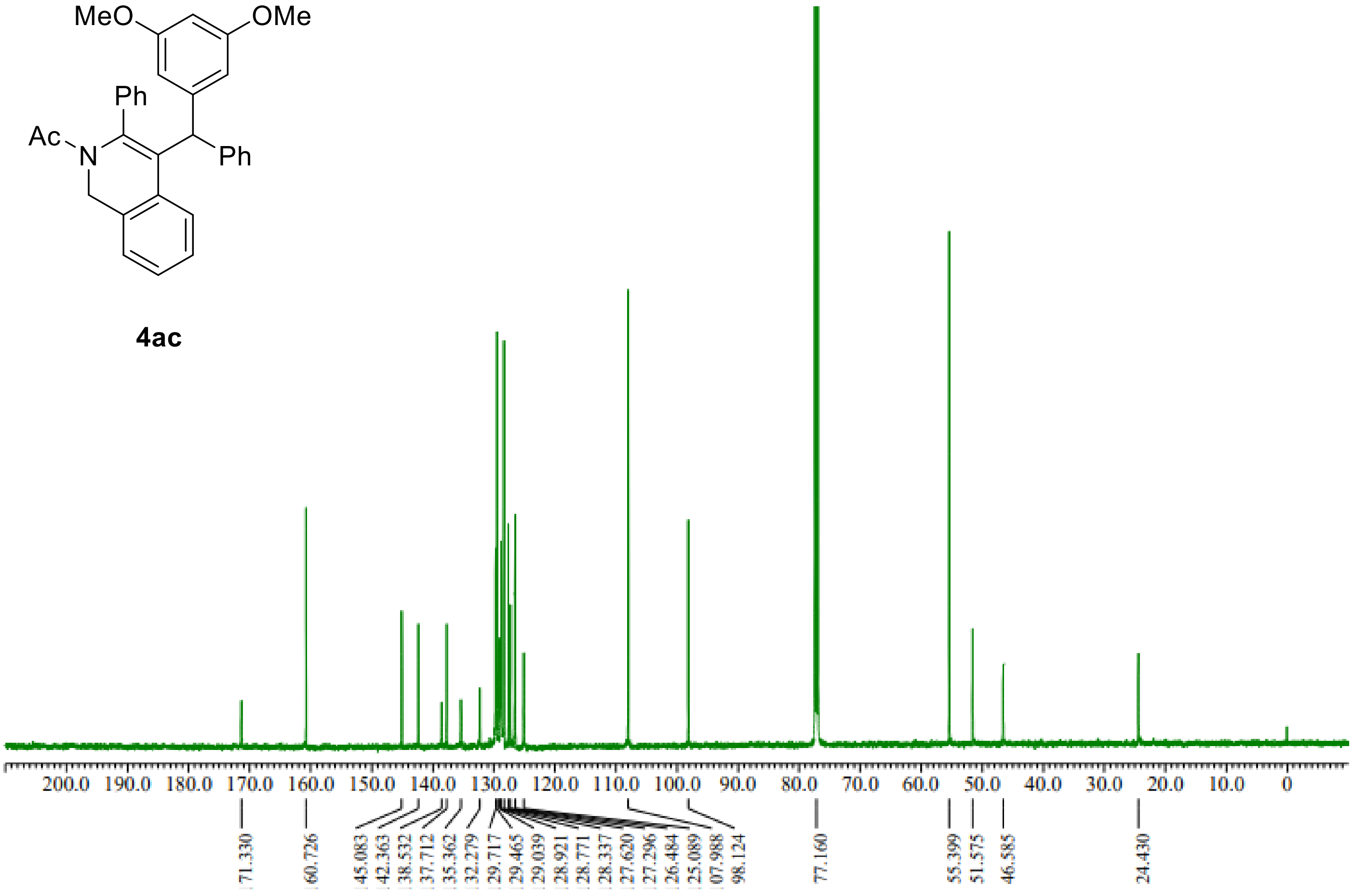

Compound **4ad**, ^{1}H-NMR (500 MHz, $CDCl_3$)

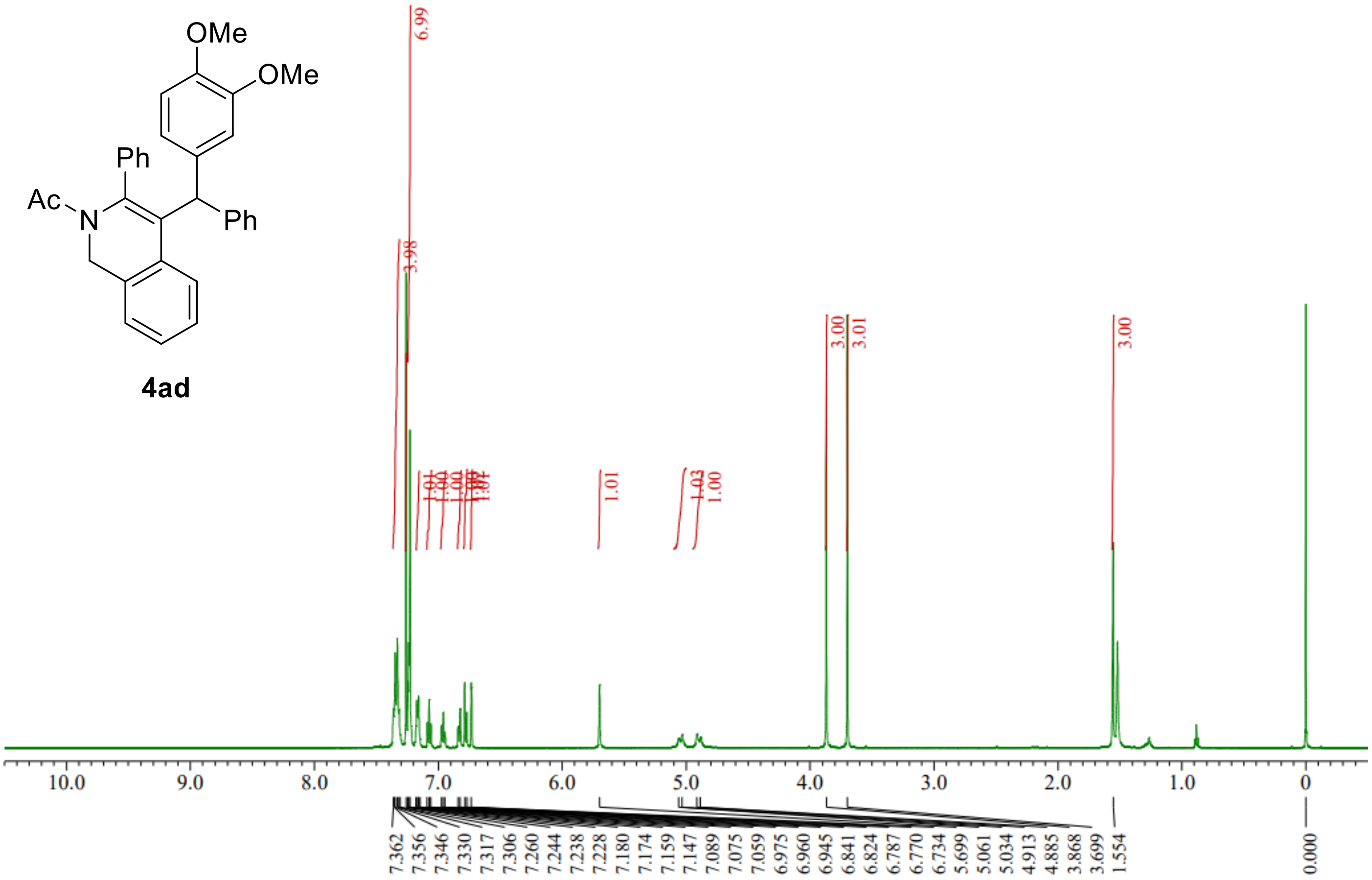

^{13}C-NMR (125 MHz, $CDCl_3$)

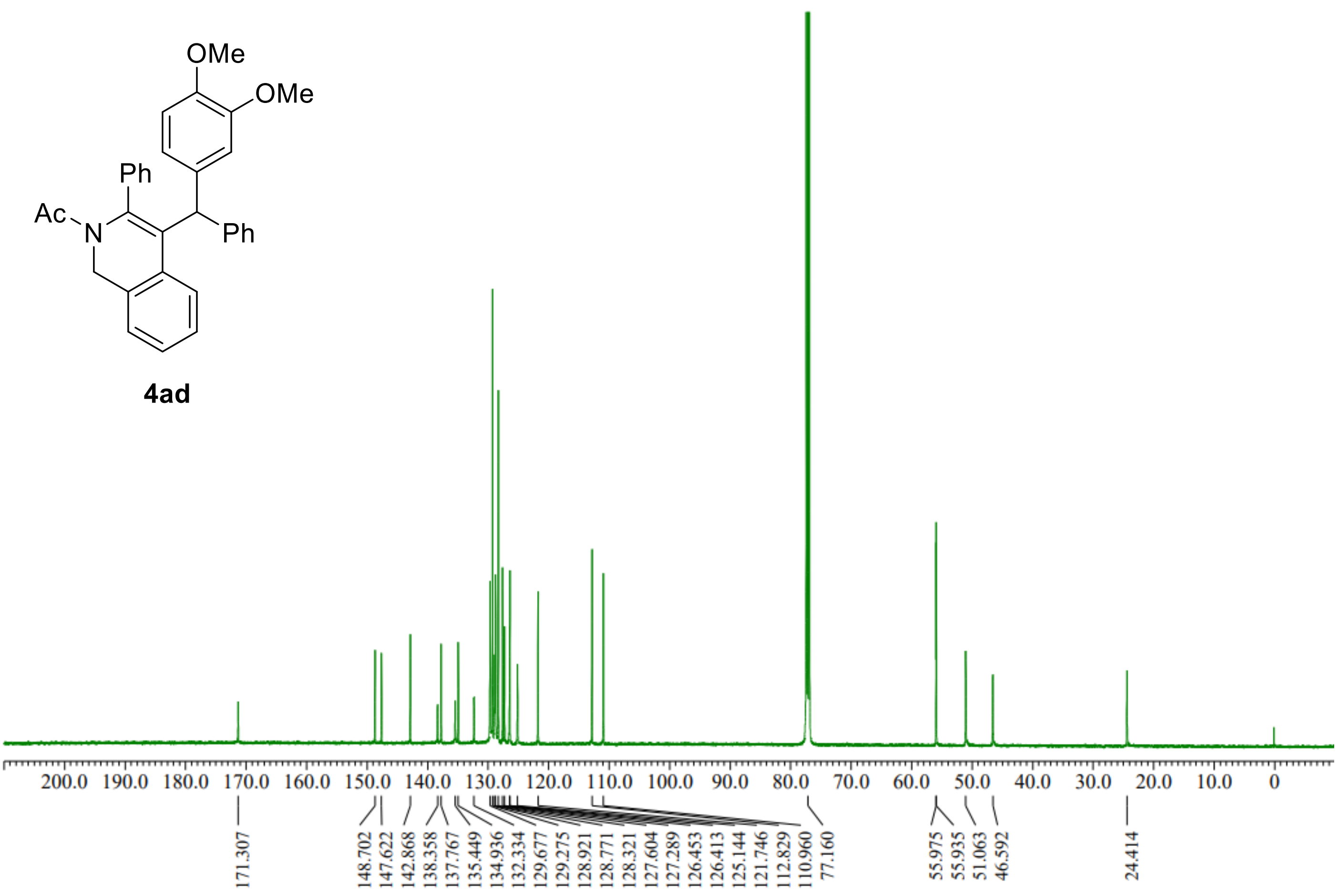

Compound **4ae**, ^{1}H-NMR (500 MHz, $CDCl_3$)

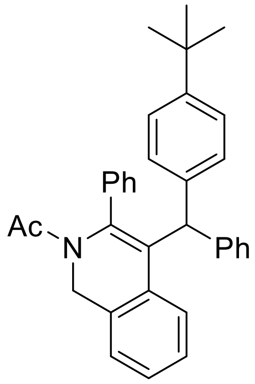

4ae

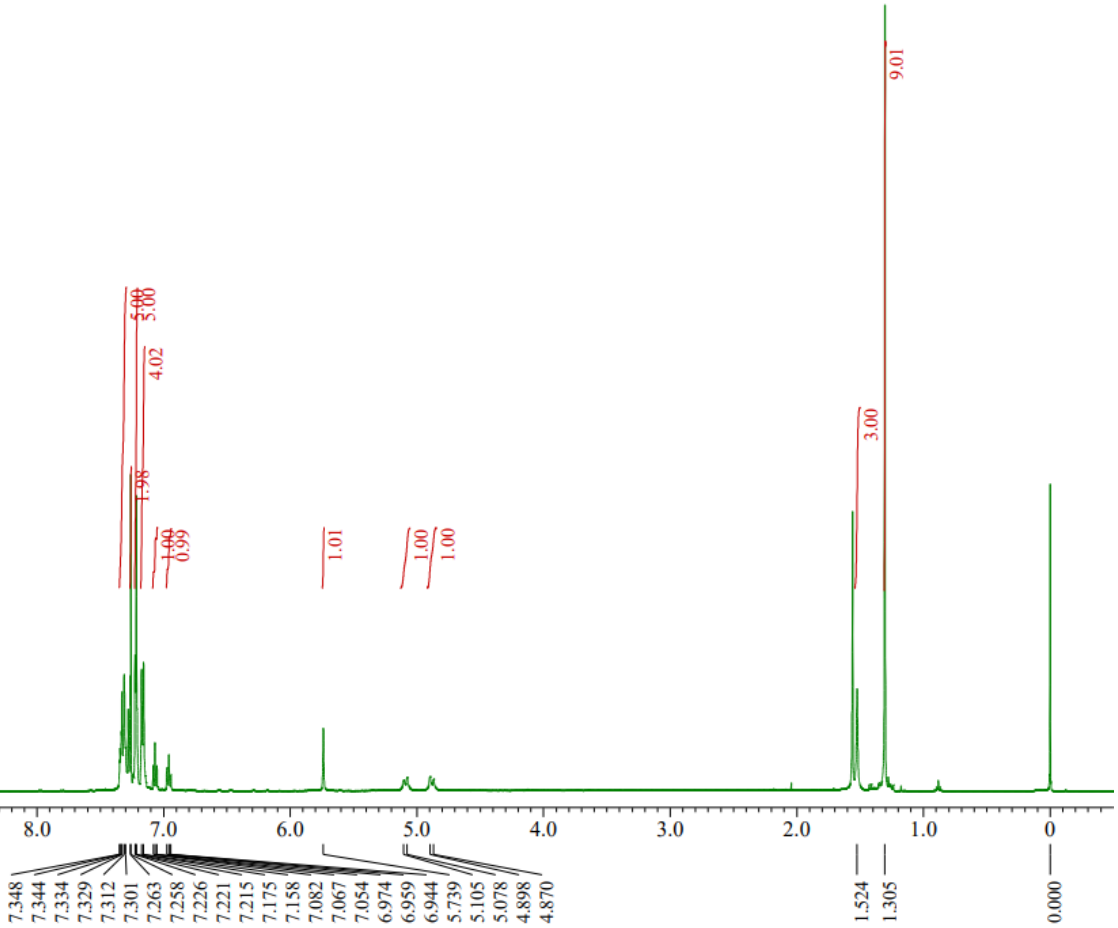

^{13}C-NMR (125 MHz, $CDCl_3$)

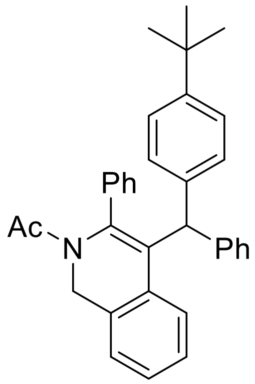

4ae

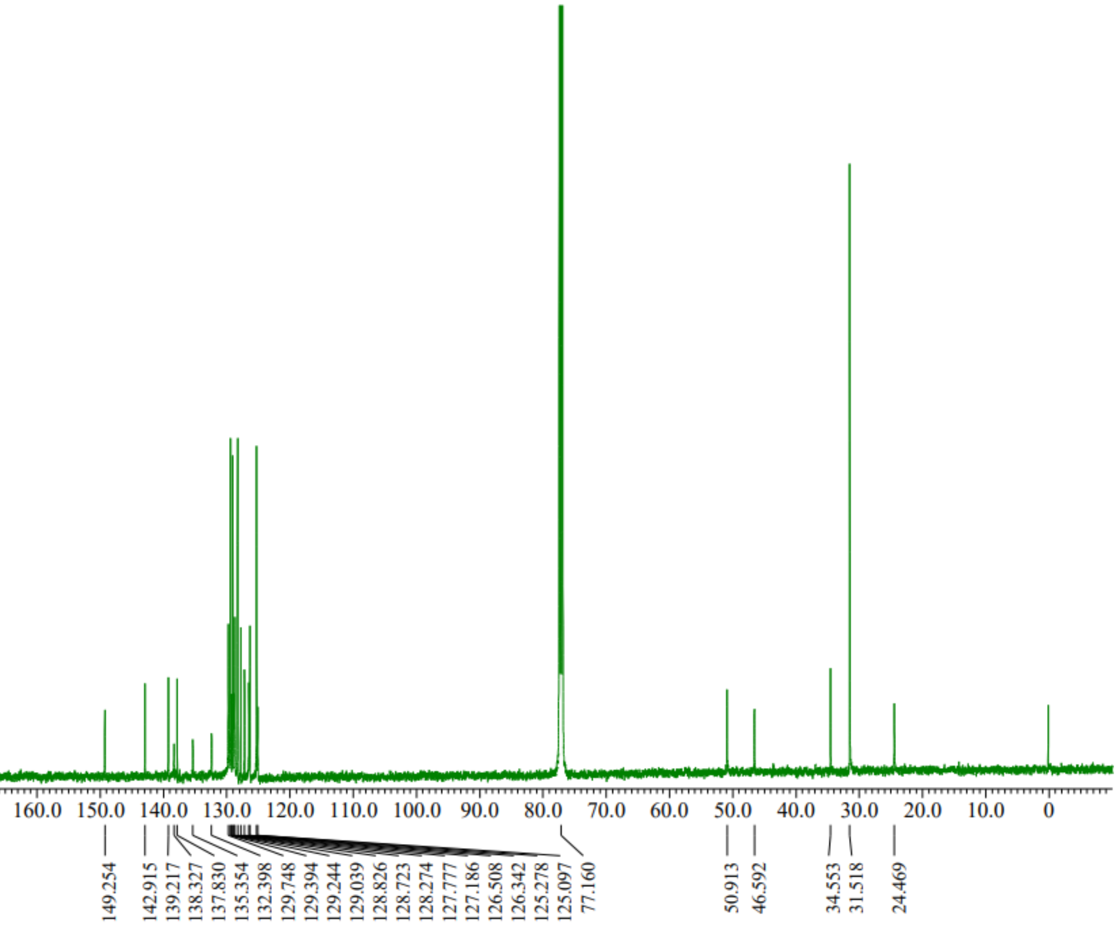

Compound **4af**, ^{1}H-NMR (500 MHz, $CDCl_3$)

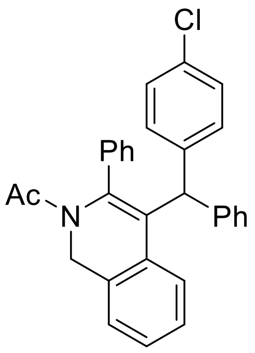

4af

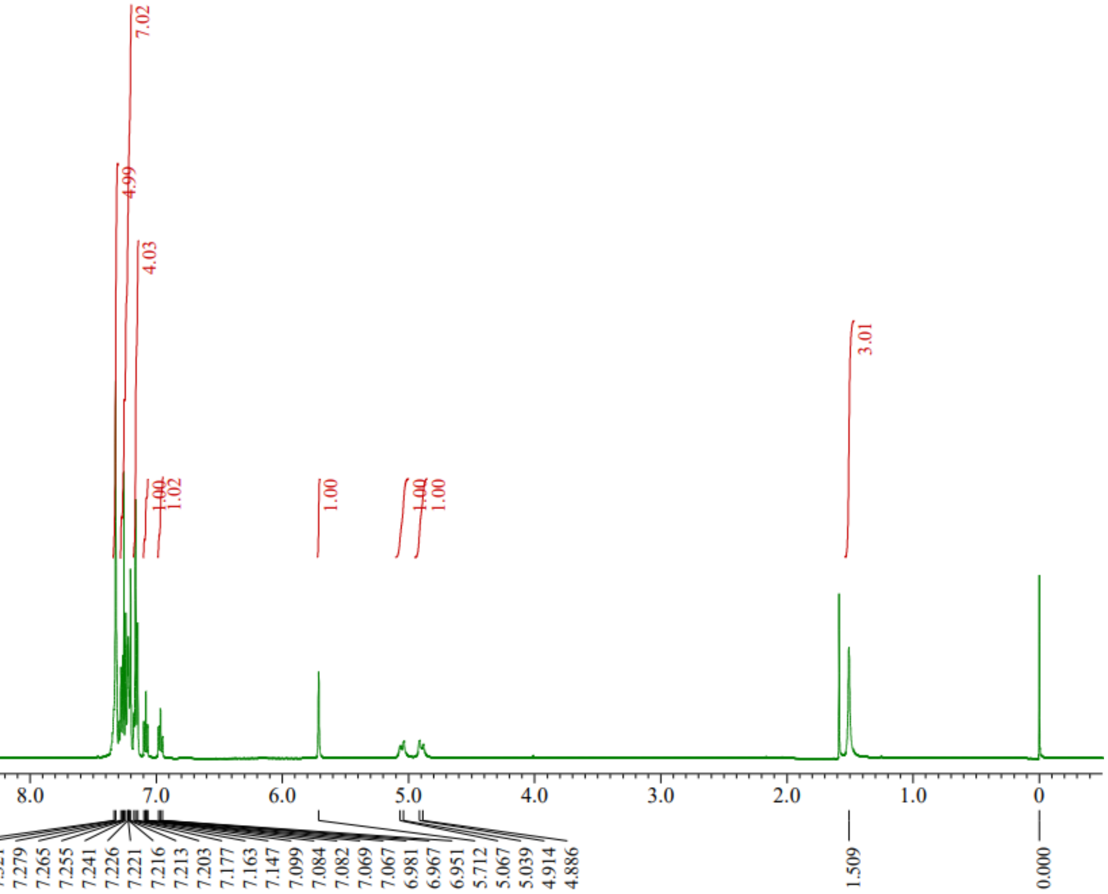

^{13}C-NMR (125 MHz, $CDCl_3$)

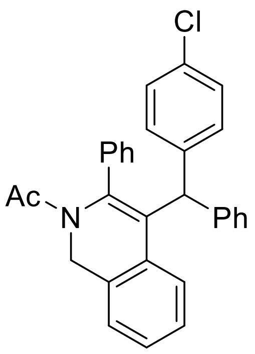

4af

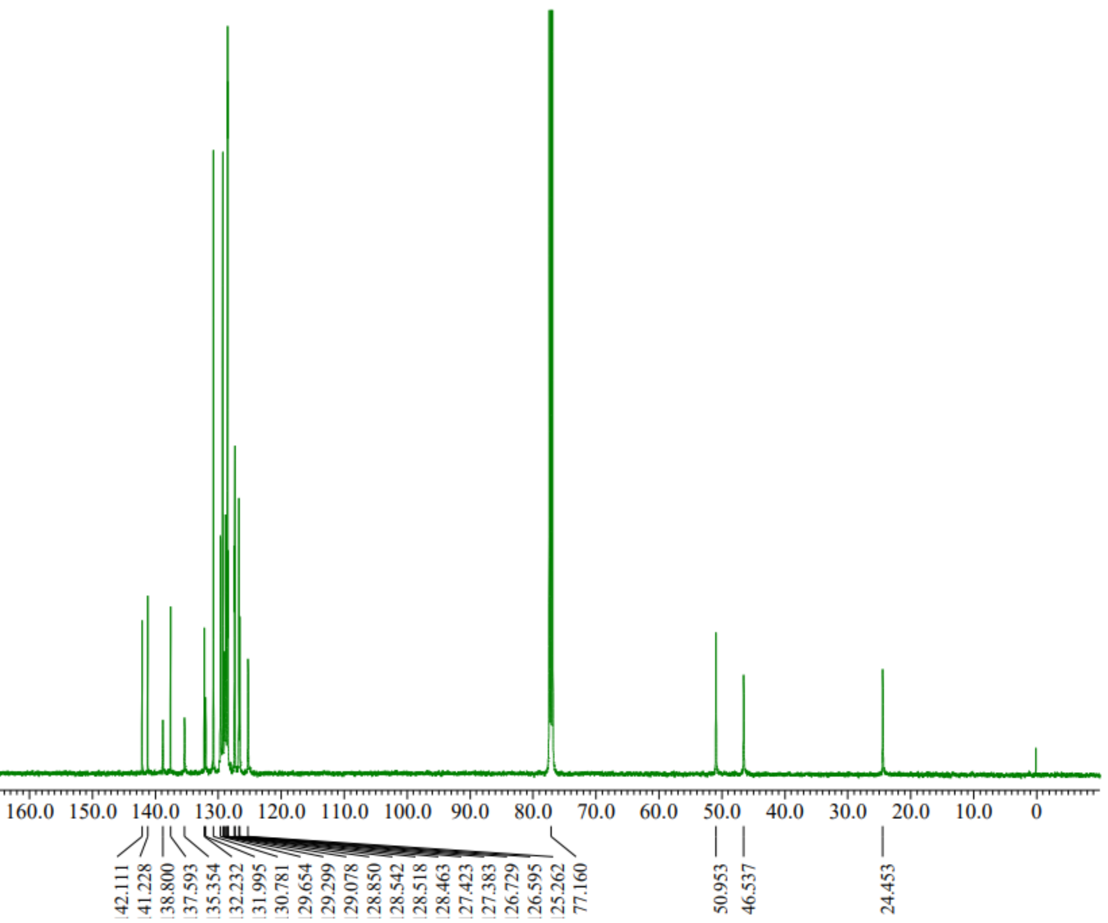

Compound **4ag (4da)**, ^{1}H-NMR (500 MHz, $CDCl_3$)

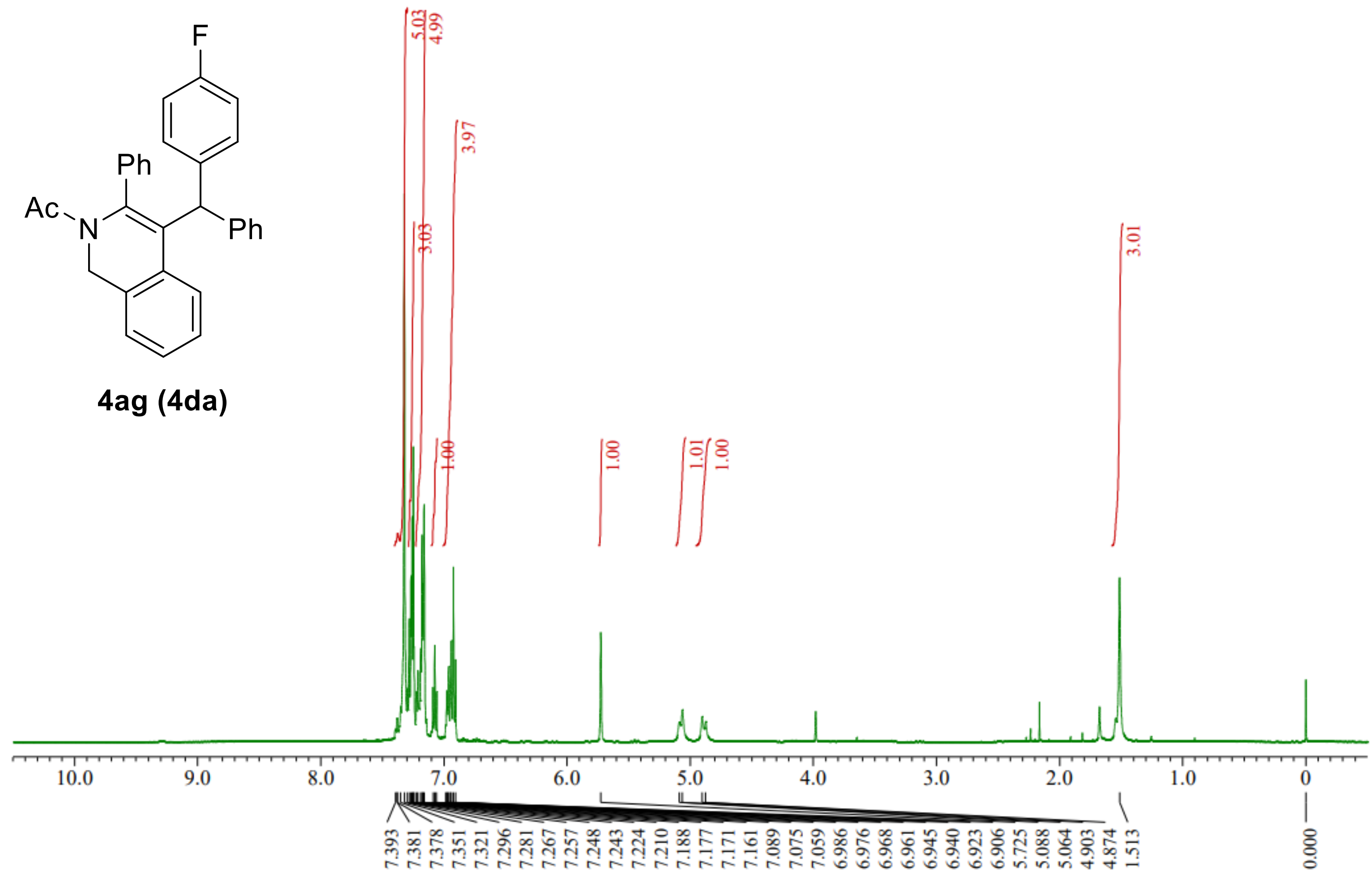

^{13}C-NMR (125 MHz, $CDCl_3$)

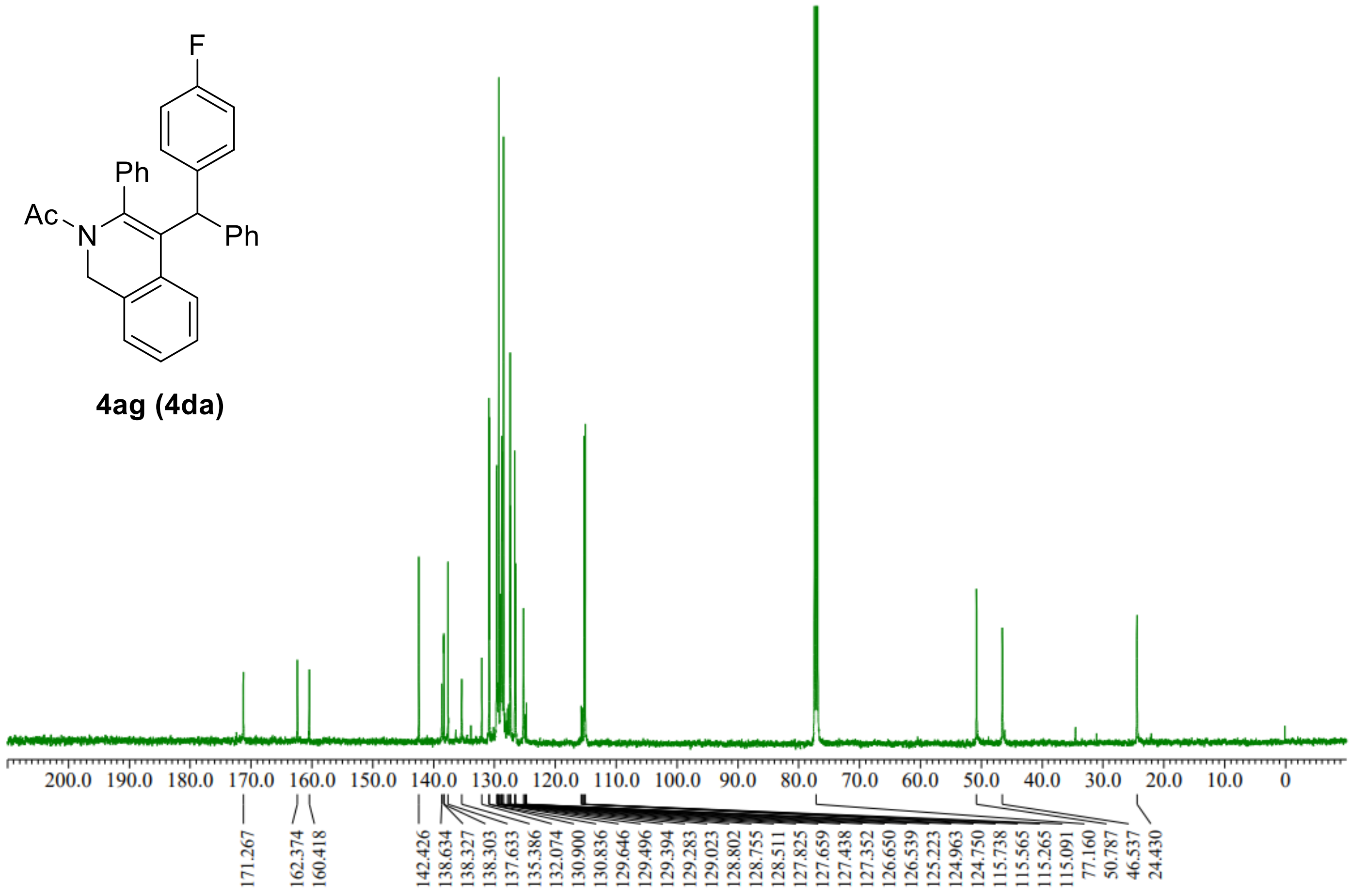

Compound **4ah**, ^{1}H-NMR (500 MHz, $CDCl_3$)

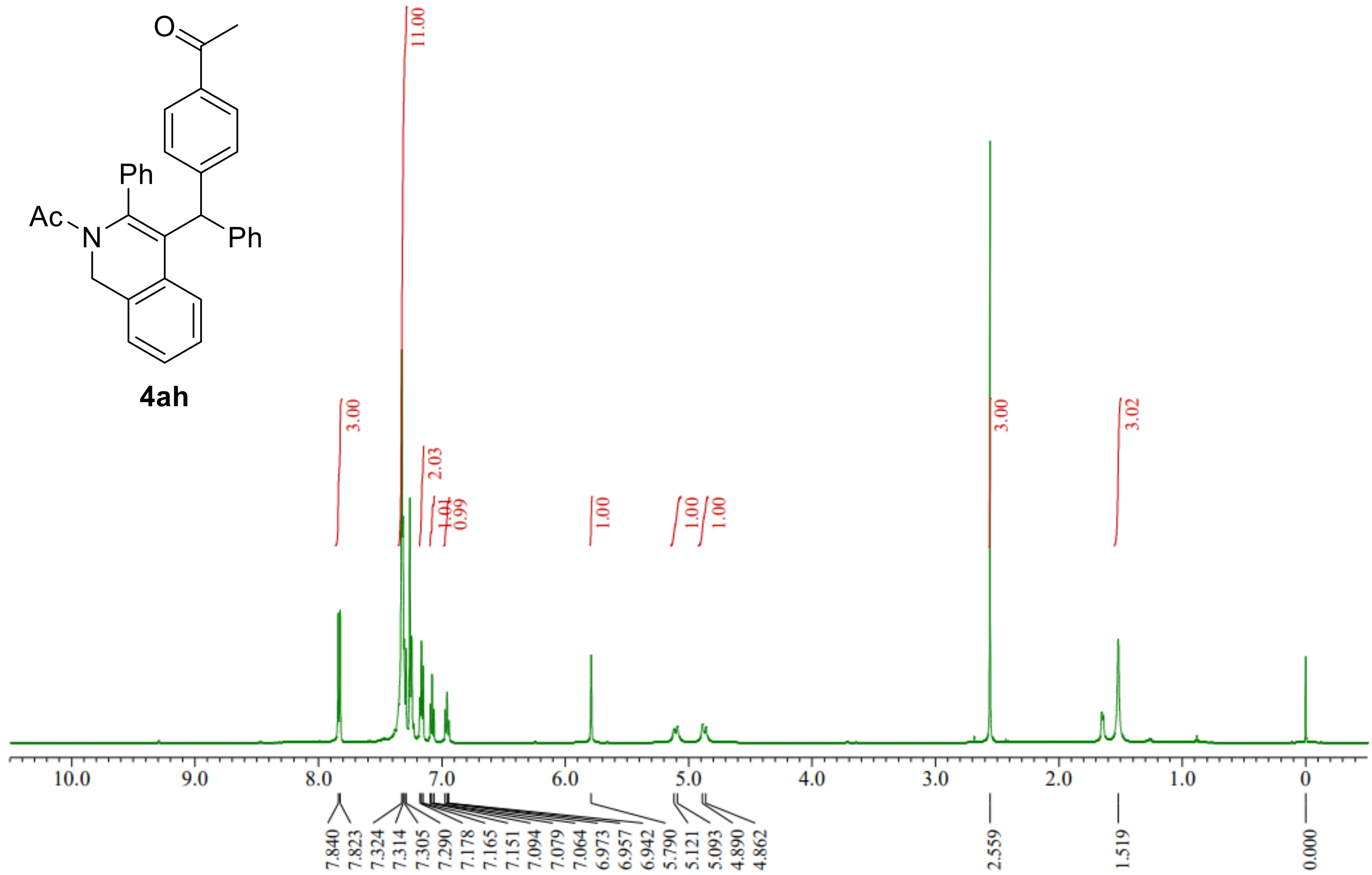

^{13}C-NMR (125 MHz, $CDCl_3$)

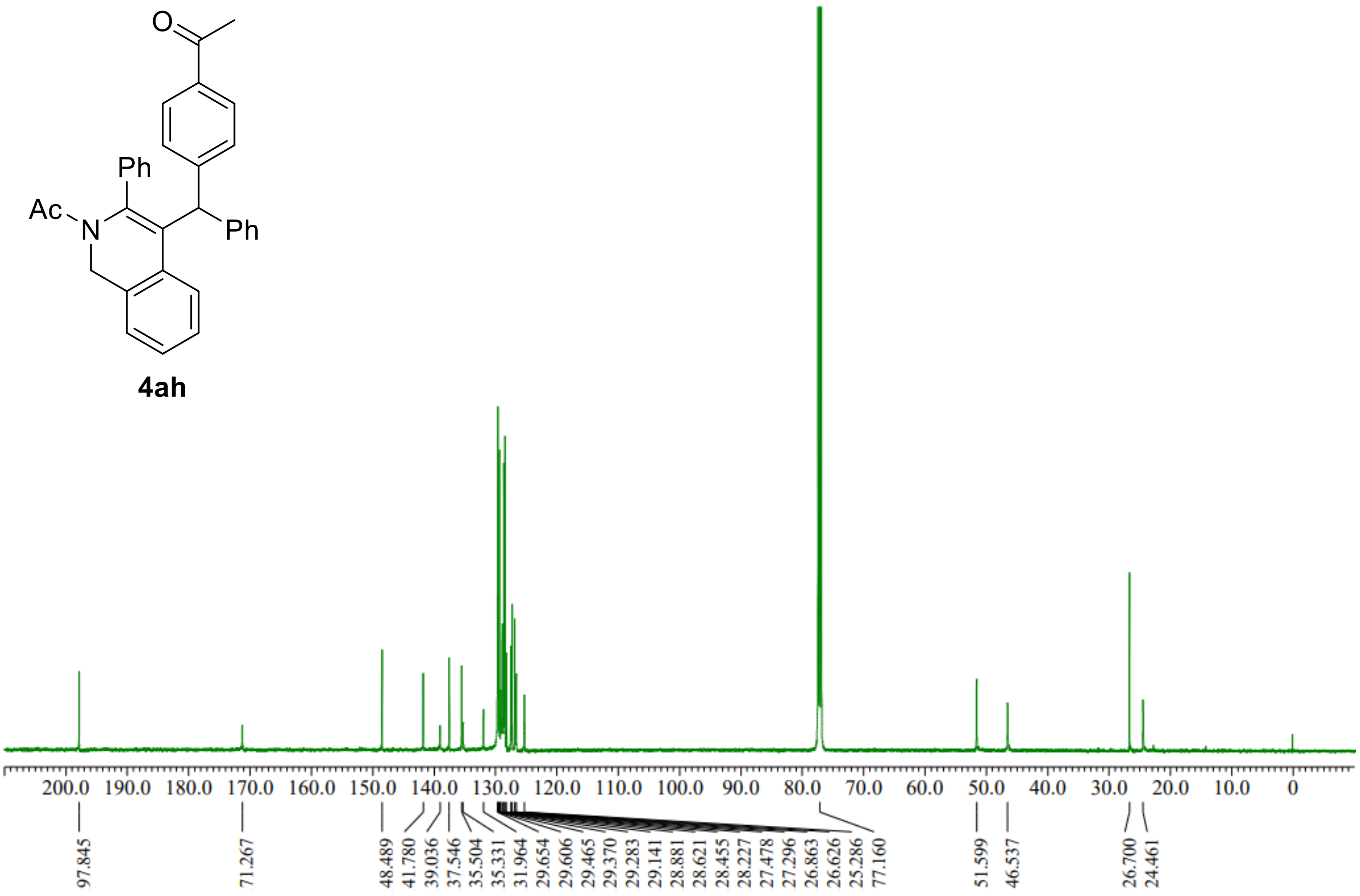

Compound **4ai**, ^{1}H-NMR (500 MHz, $CDCl_3$)

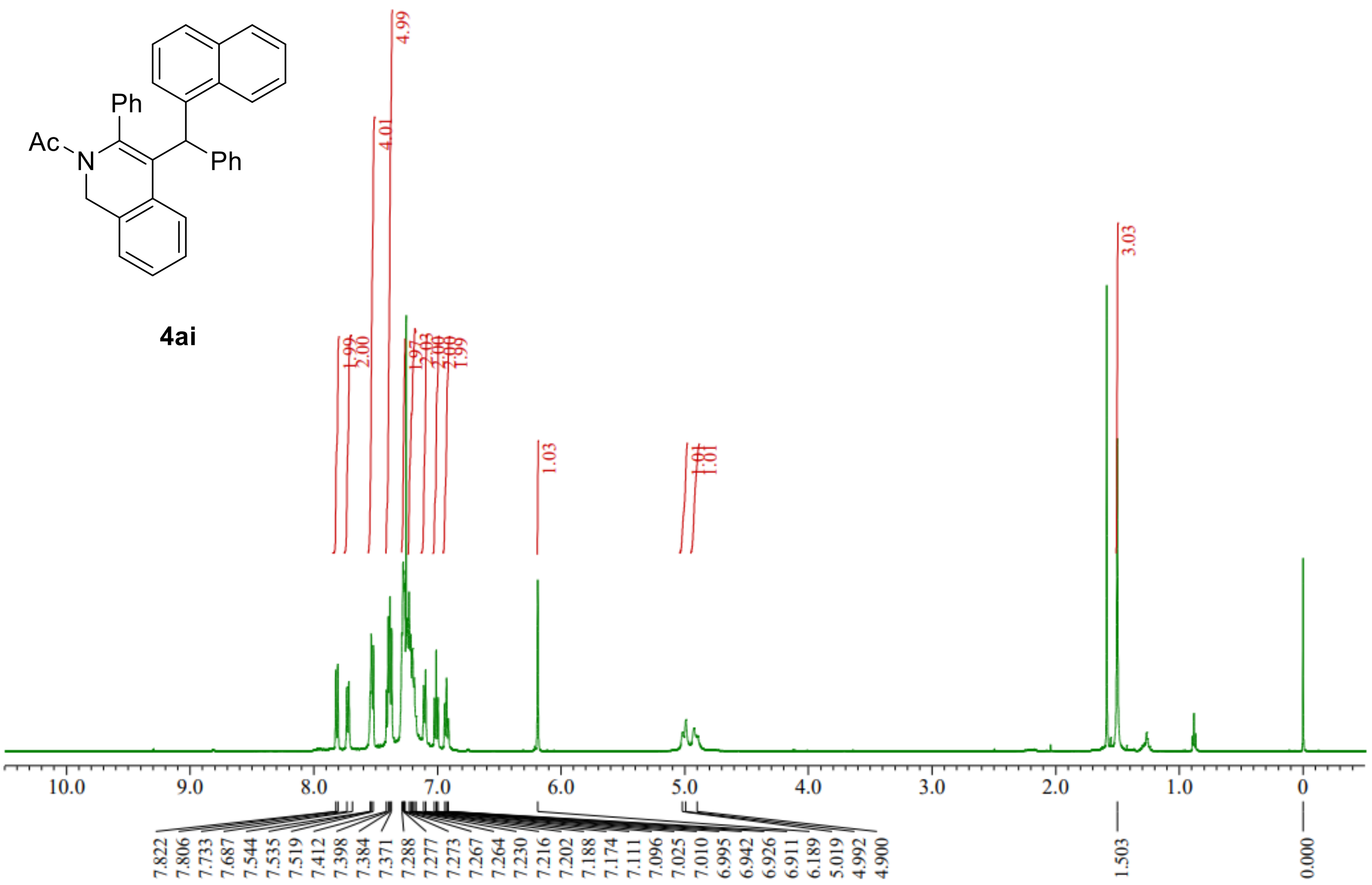

^{13}C-NMR (125 MHz, $CDCl_3$)

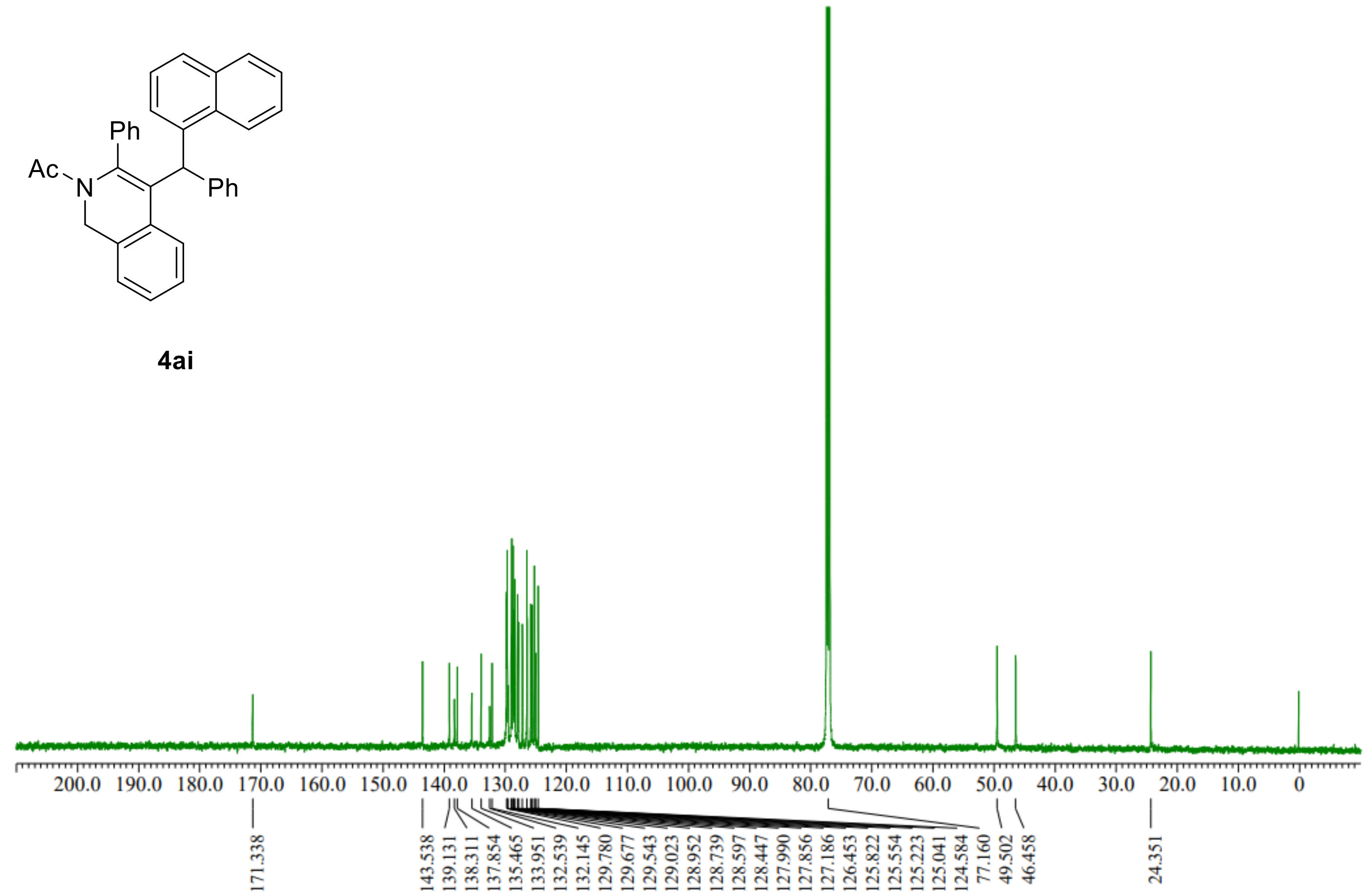

Compound **4ba**, ^{1}H-NMR (500 MHz, $CDCl_3$)

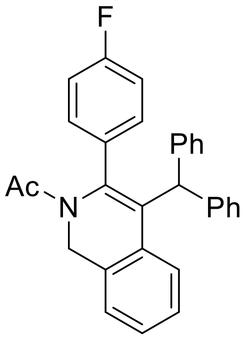

4ba

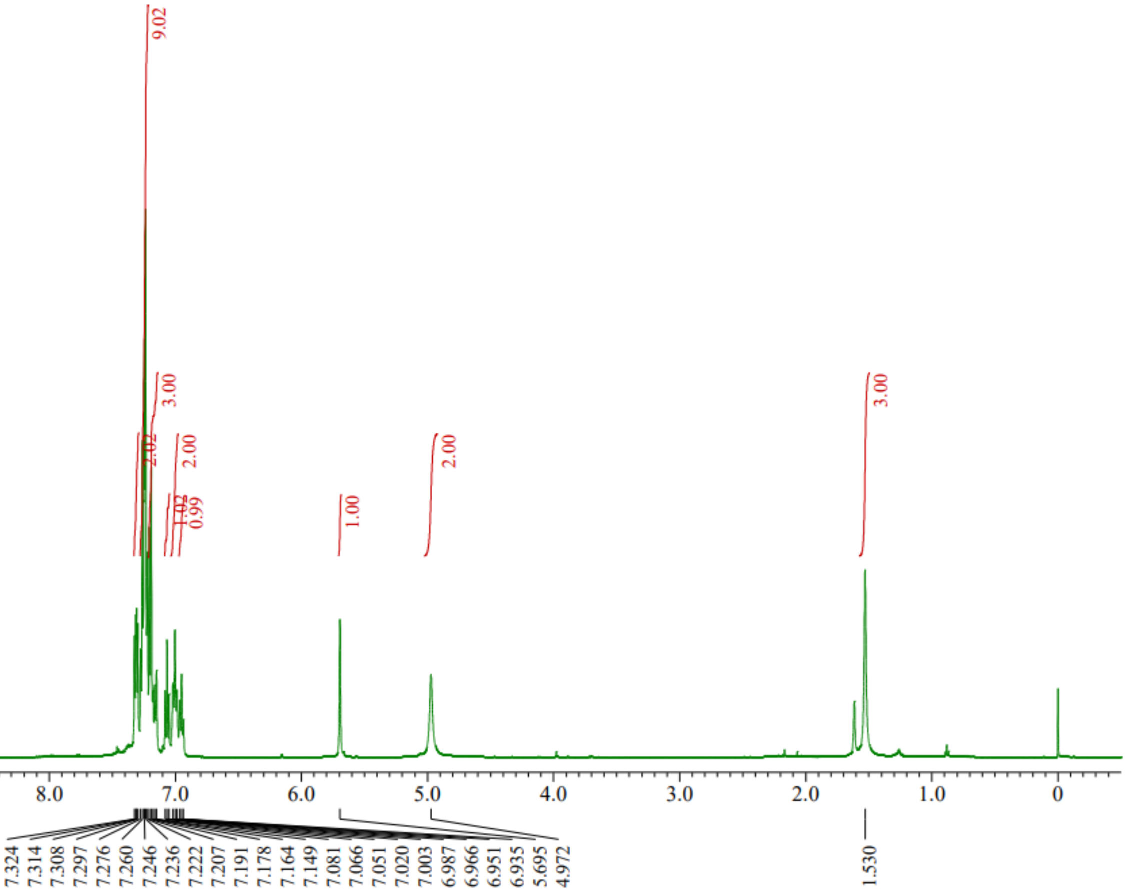

^{13}C-NMR (125 MHz, $CDCl_3$)

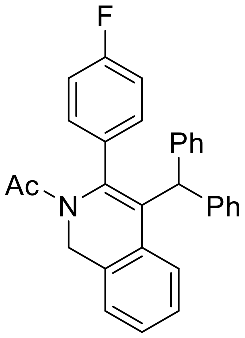

4ba

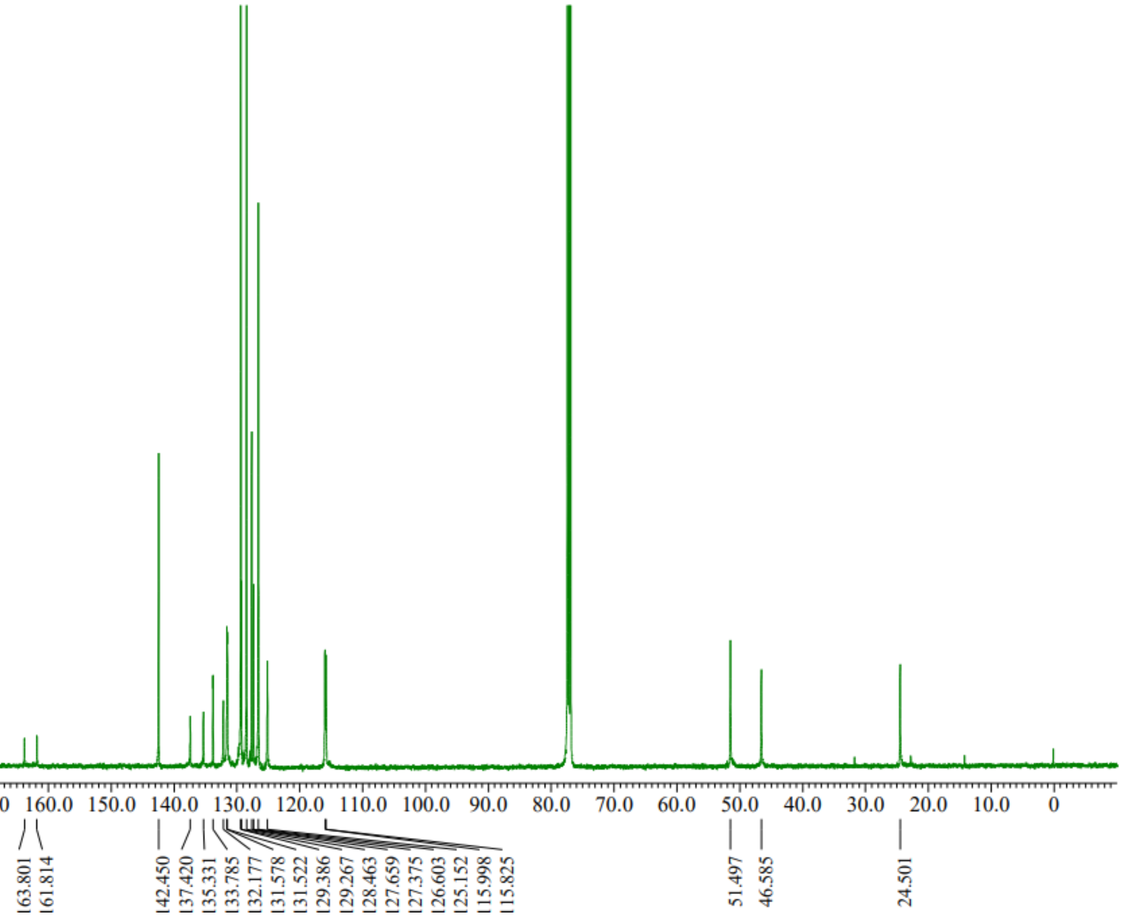

Compound **4ca**, ^{1}H-NMR (500 MHz, $CDCl_3$)

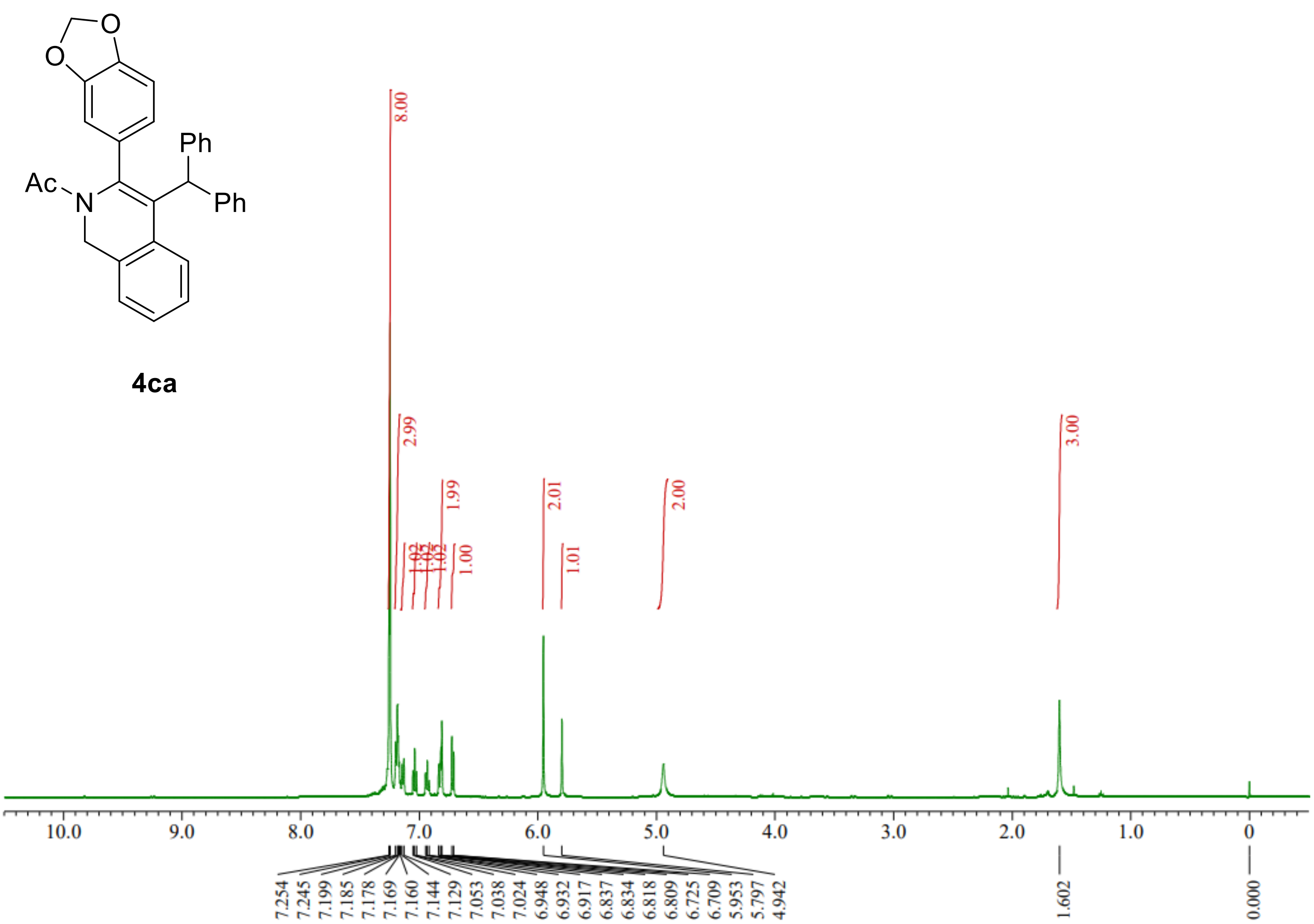

^{13}C-NMR (125 MHz, $CDCl_3$)

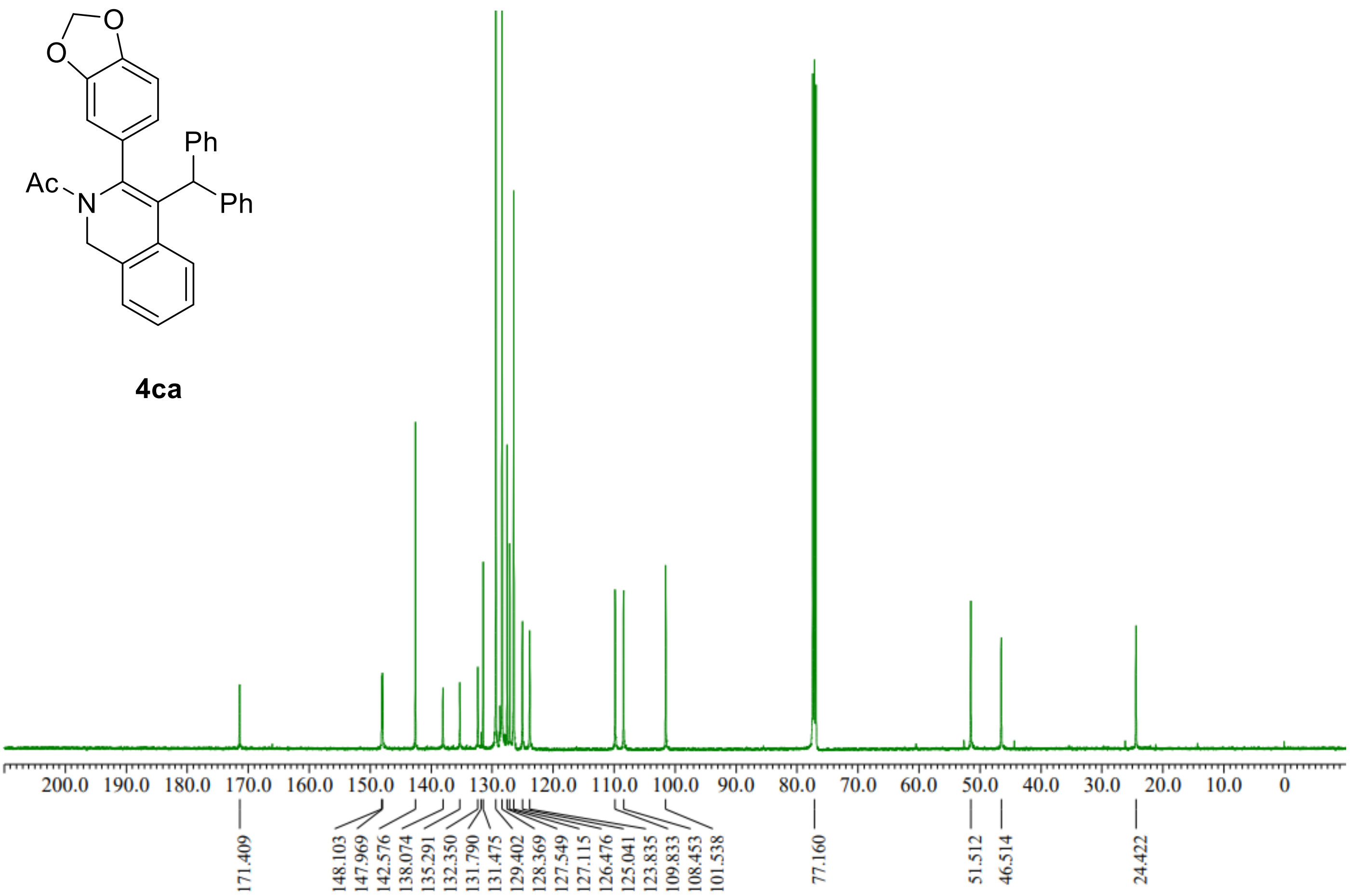

Supplement: Supplementary file 1 [file molecules-29-02917-s001.zip › molecules-3070823-supplementary.pdf]
